# Analysis of sex-based differences in energy substrate utilization during moderate-intensity aerobic exercise

**DOI:** 10.1007/s00421-021-04802-5

**Published:** 2021-09-22

**Authors:** Antonella Cano, Lucia Ventura, Gianluca Martinez, Lucia Cugusi, Marcello Caria, Franca Deriu, Andrea Manca

**Affiliations:** 1 Department of Biomedical Sciences, University of Sassari, Viale S. Pietro 43/b, 07100 Sassari, Italy; 2 Unit of Endocrinology, Nutritional and Metabolic Disorders, AOU Sassari, Sassari, Italy

**Keywords:** Energy metabolism, Exercise physiology, Sex characteristics, Aerobic exercise

## Abstract

**Purpose:**

To explore sex-based differences in energy substrate utilization during moderate-intensity aerobic exercise; to identify the underpinning candidate physiological mechanisms.

**Methods:**

Three databases were searched from inception to August 2020. Pertinent studies quantifying the utilization of substrates during moderate aerobic exercise in healthy men and reproductive-age women were considered. Studies conducted on sedentary/recreationally active and athletic populations were included and analyzed separately.

**Results:**

Thirty-five studies entered the meta-analysis (21 in sedentary/recreationally active, 14 in athletic populations). Compared to women, the respiratory exchange ratio was significantly higher both in sedentary (mean difference, MD: + 0.03; p < 0.00001) and athletic men (MD: + 0.02; *p* < 0.0001). Greater carbohydrate oxidation was observed both in sedentary (standardized MD, SMD: 0.53; *p* = 0.006) and athletic men (SMD: 1.24; *p* < 0.00001). Regarding lipid substrates, sedentary men oxidized less fat than women (SMD:  − 0.77; *p* = 0.0002), while no sex-based differences in fat oxidation were observed in athletes (SMD: 0.06; *p* = 0.77). Paucity of data prevented robust meta-analyses for protein sources. Sex hormones and different adrenergic activation were the most cited mechanisms to discuss sex-based differences.

**Conclusions:**

Meta-analyses confirmed that men display greater reliance on carbohydrates while women rely more on lipids to sustain moderate aerobic exercise. The latter finding was not confirmed in athletes, a novel aspect of the present study. Mechanistically driven research is needed to further dissect the physiological underpinnings of sex differences in substrate utilization during aerobic exercise, especially for proteins, which are still less investigated than other substrates.

## Introduction

Sex-based differences are well known to exist in endurance performance where, relative to body mass and composition, females would outperform males during exercise at submaximal intensities (Hunter et al. 2014). Women, when exercising at matched intensity, display reduced muscle fatigability and metabolic advantage in comparison to men. This fact has been attributed to a higher lipolytic efficiency (Bergström and Hultman 1966) and to a greater relative distribution and activation of fatigue resistant slow twitch fibers (Zierath and Hawley 2004; Hunter 2014; Temesi et al. 2015; Tiller et al. 2021). Nonetheless, histological, enzymatic, and hormonal aspects must be considered for the true sex-based differences in performance and fatigability, in addition to psychological and sociological factors, which could also have a confounding effect.

### Sex-based differences in carbohydrate metabolism

Sex hormones are considered key biological contributors to sex-based differences in substrate utilization. Both estrogen and progesterone alter metabolic responses, displaying opposed effects (Oosthuyse and Bosch 2010): while the former appears to impede glucose kinetics, the latter seems to potentiate it (D'Eon et al. 2002). Indeed, estrogen promotes endurance performance by hepatic glycogen sparing (Friedlander et al. 1998; Carter et al. 2001; Devries et al. 2007). High concentrations of estrogen (e.g., in the luteal phase of eumenorrheic women) can reduce reliance on muscle glycogen during moderate exercise (D'Eon et al. 2002), promoting insulin sensitivity.

One study on eumenorrheic women compared estrogen versus estrogen *plus* progesterone pharmacological administration and demonstrated higher total carbohydrate oxidation and muscle glycogen utilization for the latter condition (D'Eon et al. 2002). Controversially, data obtained in the luteal phase (when progesterone predominates) have shown lower muscle glycogen utilization during exercise in comparison with the follicular phase (when estrogen predominates) (Hackney 1999; Devries et al. 2006). The influence of progesterone alone on substrate utilization during endurance exercise is still uncovered.

### Sex-based differences in lipid metabolism

Several investigations, conducted both in sedentary and recreationally active individuals, confirmed greater reliance on lipids in women, during aerobic exercise. Such evidence indicates that not only women oxidize significantly more lipids than men (Horton et al. 1998; McKenzie et al. 2000; Lamont et al. 2001a; Henderson et al. 2007; Tarnopolsky et al. 2007; Cheneviere et al. 2011; Dasilva et al. 2011; Isacco et al. 2012; Isacco et al. 2020), but they also use less carbohydrate and protein substrates to sustain moderate exercise (McKenzie et al. 2000; Tarnopolsky 2000; Lamont et al. 2001a, 2003; Devries 2016). Comparable findings have been obtained also in athletic, endurance-trained populations (Phillips et al. 1993; Knechtle et al. 2004; Riddell et al. 2003; Wallis et al. 2006).

During exercise, the greater mRNA expression of genes associated with free fatty acid (FFA) transport to plasma and mitochondrial membranes in females has been associated to facilitate lipid metabolism (Kiens et al. 2004; Monaco et al. 2015) and higher lipid oxidation rate (Venables et al. 2005; Chenevière et al. 2011). Whether increased lipid metabolism in women during exercise is consequent to predominant oxidation of either plasma FFA or intramyocellular lipids (IMCL) is debated (Devries 2016). Indeed, while women display significantly larger storages of IMCL than men (Roepstorff et al. 2002; Devries et al. 2007), experimental evidence is inconclusive on whether they also have greater capacity to use this substrate.

Sex-based studies examining catecholamines’ effects on lipolysis, at rest, reported similar plasma concentrations and adipose tissue lipolytic sensitivity (Jensen et al. 1996; Millet et al. 1998). Different patterns of adrenergic receptor activation might be responsible for the diverse lipolysis regulation in men and women during endurance exercise (Hellström et al. 1996; Boschmann et al. 2002). Specifically, moderate-intensity exercise activates both β1 (lipolysis-activating) and α2 (lipolysis-inhibiting) receptors in men, whereas it activates only β1 receptors in women (Blatchford et al. 1985; Arner et al. 1990; Davis et al. 2000).

While sex differences in carbohydrate and lipid metabolism during exercise have been extensively investigated, few and controversial data are available for protein metabolism. Some authors reported significantly larger utilization of protein sources in men than women (Phillips et al. 1993; Lamont et al. 2001a), while others failed to detect any sex-based differences (Horton et al. 1998).

### Controversies and potential weaknesses in the existing literature

Several controversial findings can be traced in the available sex-comparative literature regarding the type of substrate used to sustain submaximal endurance exercise. For instance, Ruby and colleagues (2002) did not detect sex-based differences in total fat oxidation but, after data correction for body mass, fat oxidation rates were higher in men than women (Ruby et al. 2002). A highly controlled study reported greater adipose tissue triglyceride lipolysis and larger plasma FFA availability and oxidation in women than men, who were matched for percent body fat and aerobic fitness. However, the same study showed a similar total fat oxidation due to a reciprocal decrease in the oxidation rate of non-plasma-derived FFA in women (Mittendorfer et al. 2002). In line with these observations, previous studies conducted in untrained men and women with similar aerobic fitness and body fat found minimal or no difference in lipid oxidation rates (Costill et al. 1979; Powers et al. 1980; Keim et al. 1996; Horowitz and Klein 2000). Overall, body composition seems to play a role in the pattern of substrate oxidation during exercise, as the basal larger percent body fat in women would prompt a higher regional lipolysis (Davis et al. 2000; Cheneviere et al. 2011). Poor control of this parameter may be responsible for magnifying the sex-based differences in lipid oxidation rates generally reported.

Inconsistencies among the findings may be attributed also to poor control of training and nutritional status, to diverse methods employed to evaluate the metabolic rates, and different populations studied. Moreover, superficial characterization and consideration of the menstrual cycle phases, hormonal profile, and exogenous manipulation might lead to heterogenous female population.

The underpowered sample size of the studies threatens the validity of the findings, since results are subject to selection, information, and confounding biases, which are often poorly controlled in observational research (Grimes and Schultz 2002;; Simunovic et al. 2009). The precision and accuracy of estimates reported in individual studies can be significantly enhanced by grouping individual works and pooling their data via meta-analytic approaches, provided that the inherent heterogeneity across studies is controlled.

Despite the considerable number of reports on sex-based differences in energy substrate utilization during moderate-intensity aerobic exercise, there are no synthesis works, of which we are aware, that have quantitatively examined pooled data from the pertinent literature. Additionally, such body of knowledge has not been scrutinized yet in terms of its methodological quality and the risk for biases potentially threatening this literature.

Based on the above background and rationale, we performed a meta-analytic aggregation of data from sex-comparative studies to: (1) verify the extent of sex-based differences in carbohydrate, lipid, and protein metabolism during moderate-intensity aerobic exercise; (2) qualitatively appraise, code, and count the physiological mechanisms underpinning differences in substrate utilization between men and women; (3) further explore whether sex-based responses to exercise and putative mechanisms differ depending on the training status.

## Methods

The Preferred Reporting Items for Systematic Reviews and Meta-Analyses (PRISMA) guidelines and flowchart diagram were used as a reporting structure for this meta-analysis (Liberati et al. 2009).

### Selection of studies

The following databases were searched to retrieve pertinent articles: PubMed (including Medline), Scopus, and Web of Science. The search combined keywords, Medical Subject Headings (MeSH) and matching synonyms relevant to the topic (metabolism OR lipids metabolism OR carbohydrates metabolism OR glycogen metabolism OR glucose metabolism OR energy metabolism OR energetic metabolism OR protein metabolism) AND Oxygen Consumption/physiology [MeSH] AND Physical Endurance/physiology [MeSH] AND (male AND female) AND (gender OR sex). Only case–control, cross-sectional, and *pre–post* studies carried out in healthy adults (18 years or older) were selected. Animal studies were excluded.

Each database was searched from the earliest available record up to August 31, 2020. To be eligible for consideration, studies had to meet the following four main criteria: (1) having determined the metabolic rate of at least one energy substrate, either raw or normalized, during endurance exercise lasting from a minimum of 30 min (to avoid missing lipid oxidation, which is negligible in the early phase of exercise; Spriet, 2014) to a maximum of 120 min (to avoid ultra-endurance exercise); (2) having tested subjects during aerobic exercise carried out at moderate intensity (between 45 and 65% of the laboratory-determined peak O_2_ consumption, according to the American Heart Association Guidelines; Fletcher et al. 2001); (3) having enrolled both healthy men and reproductive-age women, and (4) having reported, compared, and interpreted data based on sex.

Studies conducted on both sedentary/recreationally active subjects and athletic population were considered for this study. However, data were kept separated in the analysis, to avoid heterogeneity.

The initial search was undertaken by three of the authors (AM, GM, MC). The retrieved items were handled using Mendeley Desktop (Version 1.19.5, Mendeley Ltd). The titles and abstracts of the retrieved studies were then independently assessed by three authors (AC, LV, LC); duplicates and records that were clearly ineligible/out of scope were excluded at this stage. When the title or abstract presented insufficient information to determine eligibility, the full-text papers were evaluated. Based on the information presented in the full manuscripts, eligible studies were included in the qualitative analysis. In cases of disagreement, consensus was reached by discussion and, if necessary, the opinion of a fourth author (AM) was sought (in five occasions) to reach the final decision. When the set of included articles was completed, all their reference lists were manually checked for further relevant publications by three of the authors (AC, LV, LC). Articles including mixed population (i.e., enrollment of both recreationally active and athletes, without reporting data separately) or presenting sex imbalance (e.g., enrollment of more males than females) were not included in the meta-analysis, to control inherent heterogeneity across the studies.

### Assessment of study quality, risk of bias and overall quality of the evidence

The included studies were assessed independently by three authors (AC, LV and LC) for methodological quality and risk of bias, employing the Study Quality Assessment Tools of the National Institutes of Health (https://www.nhlbi.nih.gov/health-topics/study-quality-assessment-tools). Specifically, the “Quality Assessment Tool for Before–After (Pre–Post) Studies with No Control Group” was employed. This tool consists of a set of 12 criteria in the form of questions covering the main sources of bias. Satisfying 75–100% or 25–75% or < 25% of the criteria is indicative of low, moderate, or high risk of bias, respectively. In case of non-applicable criteria/questions, the total score was calculated out of the highest number of applicable items rather than out of the predefined 12 items. Disagreements between the three authors were resolved by discussion. If consensus could not be reached, the opinion of a fourth author (AM) was sought (in two occasions).

### Data extraction process and pre-planned meta-analyses

A customized data extraction form was developed and applied to each included article by one author (AM) and the extracted data were checked for accuracy by a second author (LC). The extracted data included information regarding the participants (e.g., sex ratio, fitness level, anthropometric characteristics, oral contraceptives use, dietary habits), the pre-testing condition and exercise protocol (e.g., pre-testing dietary conditions, menstrual phase, duration, intensity relative to peak O_2_ consumption, exercise modality—e.g., walking, cycling, etc.), outcome measures (i.e., raw or normalized as percentage), main findings (e.g., carbohydrates oxidation: men > women).

Based on the state of the art, we predefined a set of sex-comparative meta-analyses of percent and raw data for the following variables: carbohydrate oxidation (including, but not limited to, muscle glycogen and glucose utilization, rate of appearance and disappearance); lipid oxidation (including, but not limited to, FFA and IMCL); protein oxidation (including, but not limited to, amino acid utilization and disposal). To control for heterogeneity deriving from inconsistencies in the training status, we performed separate analyses for sedentary/recreationally active and athletic populations.

### Thematic analysis of the mechanisms mediating sex-based differences

To gather mechanistic insights into the possible physiological correlates of the observed sex differences in substrate utilization, a thematic analysis was performed. Each individual study was carefully read to outline relevant investigated and/or suggested physiological mechanisms. Original text extracts (direct quotes) were then obtained, and recurrent concepts were highlighted and subsequently coded (e.g., “adrenergic mechanism: receptor type and catecholamines levels” or “adrenergic regulation of lipid mobilization”). Single themes that could gather several codes (e.g., “adrenergic activation”) were generated a posteriori by consensus among the three authors (AC, AM, LV). Themes were then highlighted within each paper and used to qualitatively appraise the mechanisms investigated and/or suggested by the authors. In case the authors tested or proposed more than one mechanism, only those for which sex-based differences emerged were computed. Mechanisms associated to both fat and carbohydrate metabolism were considered separately. If two or more mechanisms were found/suggested to mediate the observed differences between men and women, the hierarchical order of importance drawn by the authors was followed.

### Data analysis

A meta-analysis was performed if at least three studies reported data for the same outcome measure. RevMan 5.4.1 software (Review Manager, The Cochrane Collaboration; 2020) was used to aggregate the extracted data and to obtain pooled estimates of the difference between men and women. Raw data (means and standard deviations, SD) were extracted or calculated from other statistics reported in the paper (i.e., standard error; 95% confidence interval, CI). If studies reported outcomes exclusively through graphs, the mean scores and the related measures of spread (SD, standard error, 95% CI) were estimated employing GetData Graph Digitizer (version 2.26.0.20). A random-effects model was chosen for all meta-analyses to account for potential methodological differences in the assessment and training protocols across studies, as conventionally done in biomedical research (Borenstein et al. 2010). To allow interpretation of the pooled estimate of an effect, the weighted mean difference (MD) with 95% CI was calculated when pooling data from an outcome measure that was homogeneously assessed across studies, whereas the standardized mean difference (SMD) was calculated when the extracted data for one outcome were expressed with different measurement units, or when different testing protocols or exercise modalities (e.g., treadmill walking, over ground walking, cycle ergometer) were employed. Additionally, to estimate the magnitude of the effect size through a standardized index, the SMD was reported for all MD (taking an SMD of 0.2 as small, 0.5 as moderate, and 0.8 as large). In both cases, the level of significance was set at *p* < 0.05, as conventionally done in meta-analyses. Heterogeneity across the studies was evaluated using the Chi-square and the inconsistency (I^2^) test; a value > 50% was considered indicative of significant heterogeneity (Higgins et al. 2003). In case of heterogeneity exceeding this threshold, a *leave-one-out* sensitivity analysis was performed to check whether our findings were driven by a single study.

For those comparisons in which data were obtained from at least ten studies (Sterne et al. 2011), publication bias was assessed by visual inspection of funnel plot asymmetry. To evaluate differences in methodological quality between the studies conducted in sedentary/recreationally active and athletic populations, the Mann–Whitney *U* test was performed. As for all the other comparisons, the significance level was set at *p* < 0.05.

## Results

### Selected articles

**Fig. 1:**
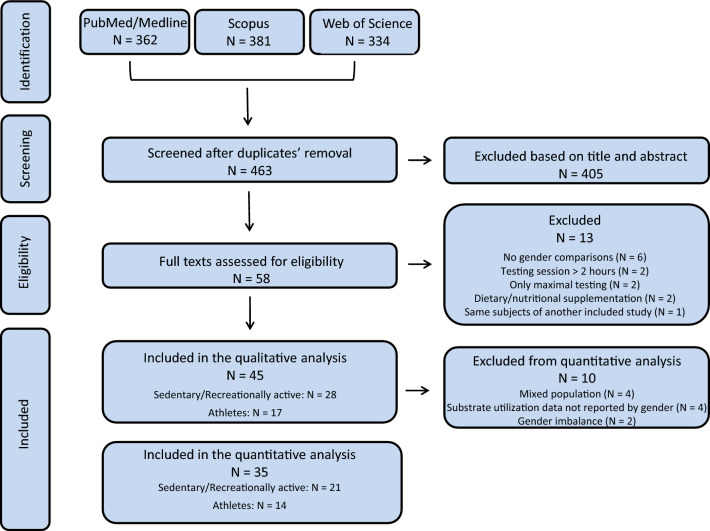
Flowchart of the studies

The search strategy identified 1077 potentially relevant records (from PubMed/Medline, 362 records; Scopus, 381 records; Web of Science, 334 records). After merging the items retrieved from the databases, duplicates were removed leaving 463 unique articles. Of these, 405 were discarded based on title and abstract, whereas 58 were assessed in full text. Thirteen studies, which did not satisfy the predefined inclusion criteria, were excluded. The remaining 45 studies, deemed eligible, were included in the qualitative analysis. Figure 1 presents the flowchart of the study selection process. The main features of the 45 studies included in the qualitative analysis are summarized in Tables 1 and 2 (i.e., participants’ status, pre-testing conditions and employed exercise protocols) and Tables 3 and 4 (i.e., outcome measures, main findings and suggested physiological mechanisms), in sedentary/recreationally active (28 studies) and athletic (17 studies) populations, respectively.

**Table 1:** Participant’s features, pre-testing and testing conditions and quality of studies carried out in sedentary or recreationally active healthy subjects and included in the qualitative analyses (*N* = 28)

Study Country	Participants	Menstrual cycle phase	Oral contraception	Diet assessment	Pre-testing condition and testing session	Study quality
Arner et al. 1990 Sweden	*N* = 17; 8 M, 9 W Recreationally active Age *(y)*: M: 32 ± 3; W: 37 ± 4 Weight *(kg)*: not reported BMI: M 24.2 ± 0.05; W: 21.5 ± 0.7 VO_2_ max: not reported	Not reported	Not reported	No diet control	Overnight fast Cycling 30 min at 65% VO_2_ max	5/9
Blatchford et al. 1985 USA	*N* = 12: 6 M, 6 W Recreationally active Age *(y):* M: 33.7 ± 1.9; W: 30.7 ± 0.8 Weight *(kg):* M: 81.9 ± 4.7; W: 65.8 ± 4.5 BMI: not reported VO_2_ max: M: 44.2 ± 3.3; W: 36.4 ± 3 ml/kg/min	Not reported	NO	Not reported	12-h fast Walking on treadmill 90 min at 35% VO_2_ max	6/9
Boschmann et al. 2002 USA	*N* = 20; 9 M, 11 W Recreationally active Age *(y):* M: 33 ± 2; W: 32 ± 2 Weight *(kg):* M: 68 ± 3, W: 62 ± 4 BMI: not reported VO_2_ max: M: 2.49 ± 0.11; W: 2.57 ± 0.20 l/min	Not reported	Not reported	No diet control	Overnight fast Cycling supine position 70 min at 50% VO_2_ max	6/9
Burguera et al. 2000 USA	*N* = 12; 6 M, 6 premenopausal W Sedentary Age *(y)*: M: 32 ± 3; W: 28 ± 2 Weight *(kg):* M: 84 ± 6.6; W: 65.4 ± 4.1 BMI: not reported VO_2_ max normalized to fat-free mass: M: 56 ± 3; W: 51.0 ± 1 ml/kg/min	Follicular (method not specified)	Not reported	Isoenergetic diet seven days before study	Unclear Cycling 90 min at 45% VO_2_ peak	5/9
Carter et al. 2001 Canada	*N* = 16: 8 M, 8 W Sedentary Age *(y):* M: 22 ± 1; W: 22 ± 1 Weight *(kg):* M: 78.1 ± 2.5; W: 66.6 ± 3 BMI: Not reported VO_2_ max: M: 41.5 ± 2.4; W: 32.3 ± 1.6 ml/kg/min	Mid follicular (blood level measurements)	Not reported	Checklist diet to consume and record the day before experimental trial	Defined formula 3 h before test session Cycling progressive exercise test at 60% VO_2_ peak	5/9
Cheneviere et al. 2011 Switzerland	*N* = 24; 12 M, 12 eumenorrheic W Recreationally active Age *(y):* M: 27.8 ± 1.1; W: 25.3 ± 1.5 Weight *(kg):* M: 75.0 ± 2.0; W: 61.7 ± 2.3 BMI: M: 23.4 ± 0.6; W: 21.5 ± 0.8 VO_2_ max normalized to fat-free mass: M: 58.5 ± 1.6; W: 55.3 ± 2.0 ml/kg/min	Early follicular (method not specified) Regular menstrual cycle reported (28.6 ± 0.8 days)	NO	No diet control	10-h overnight fast Cycling submaximal incremental test at 20%, 40%, 60%, 80%, 85% VO_2_ max	5/9
Cunningham et al. 1990 USA	*N* = 20; 9 M, 11 W Sedentary Age *(y)*: M: 33.4 ± 3.1; W: 34.9 ± 3.1 Weight *(kg):* M: 88.6 ± 4.6; W: 67.0 ± 4 BMI: not reported VO_2_ max: M: 3.12 ± 0.14; W: 1.89 ± 0.05 l/min	Not reported	Not reported	No diet control	Not reported “Exercycle” ~ 25 min, 18 sessions, 6 weeks session = 5 min warm up, cardiopulmonary segment (61.5% VO_2_ peak), 5 min cool down	5/9
Dasilva et al. 2011 Brazil	*N* = 34; 17 M, 17 eumenorrheic W Sedentary and recreationally active Age *(y)*: M: 24.0 ± 3.3; W: 22.5 ± 2.6 Weight *(kg):* M: 71.9 ± 10.1; W: 58.8 ± 6.5 BMI: M: 23.3 ± 2.2; W: 22.2 ± 1.8 VO_2_ max: M: 57.3 ± 5.9; W: 45.9 ± 5.6 ml/kg/min	Early follicular (method not specified) Normal menstrual cycle length (25–32 days)	NO	Dietary energy and macronutrient intake standardized and monitored (method not specified)	12-h overnight fast Walking on treadmill 20 min at a self-selected pace *(starting from 4.0 km/h for 2 min and then adjusted)*	6/9
Davis et al. 2000 USA	N = 16; 8 M, 8 W Sedentary and recreationally active Age *(y):* M: 29 ± 2; W: 28 ± 2 Weight *(kg)*: Not reported BMI: M: 23 ± 1; W: 22 ± 1 VO_2_ max: M: 45.0 ± 5; W: 37.0 ± 5 ml/kg/min	Mid follicular (method not specified)	Not reported	Weight maintaining diet for 3 days before study	Overnight fast Cycling 90 min at 50% VO_2_ max	6/9
Devries et al. 2007 Canada	*N* = 36; 17 M, 19 eumenorrheic W Recreationally active Age *(y):* M: 23 ± 1; W: 24 ± 1 Weight *(kg):* M: 75 ± 2; W: 62 ± 2 BMI: Not reported VO_2_ max: M: 52.0 ± 3; W: 44.0 ± 2 ml/kg/min	Mid follicular (method not specified)	YES (n = 10); NO (n = 9)	Dietary intake recorded analyzed	12 h post-absorptive Cycling 90 min at 63 ± 2% of VO_2_ peak	6/9
Devries et al. 2006 Canada	*N* = 24; 11 M, 13 W Recreationally active Age *(y):* M: 21.1 ± 1; W: 22 ± 2 Weight *(kg):* M: 80 ± 3; W: 63 ± 2 BMI: M: 25 ± 1; W: 23 ± 1 VO_2_ max: M: 45.0 ± 1; W: 39.0 ± 2.0 ml/kg/min	Follicular and luteal (menstrual cycle diary, ovulation kit for W not using OC and blood level measurements)	YES (n = 6) NO (n = 7)	The same meal on the evening before both test days	12 h post-absorptive Cycling 90 min at 65% of VO_2_ peak	6/9
Friedlander et al. 1998 USA ^#^	*N* = 18 W Sedentary Age *(y)*: W: 23.8 ± 2 Weight *(kg):* W: 63.7 ± 2.1 BMI: not reported VO_2_ max: W: 33.5 ± 1 ml/kg/min	Mid follicular (blood levels measurements) Regular menstrual cycle (28–35 days)	NO	Three-day dietary record at the beginning, 4weeks into training, and before each post-training isotope trial Twenty-four hour dietary intake preceding each of the four isotope trials	Dinner (12 h) selected and repeated before each trial. Standardized snack before bed (eight–ten hours), standardized breakfast (one–two hours) before reporting to the laboratory. Post-absorptive Cycling continuous, progressive maximal stress test 60 min from 50 to 75% VO_2_ peak	5/9
Friedlander et al. 1999 USA ^#^	*N* = 20 M Sedentary Age *(y)*: M: 25.5 ± 0.7 Weight *(kg):* M: 78.6 ± 2 BMI: not reported VO_2_ max: M: 46.5 ± 1.1 ml/kg/min	Not applicable	Not applicable	Twenty-four dietary intake preceding each of the four isotope trials	Dinner (12 h) selected and repeated before each trial. Standardized snack before bed (eight–ten hours), standardized breakfast (one–two hours) before reporting to the laboratory. Post-absorptive Cycling continuous, progressive maximal stress test 60 min from 50 to 75% VO_2_ peak	5/9
Hellström et al. 1996 Sweden	*N* = 28; 14 M, 14 W Recreationally active Age *(y)*: M: 32.6 ± 2; W: 35.8 ± 3 Weight *(kg)*: Not reported BMI: M: 23.5 ± 0.46; W: 22.7 ± 0.68 VO_2_ max: not reported	Not reported	Not reported	Standard Swedish diet	Overnight fast Cycling 30 min at 2/3 of their max working capacity	6/9
Henderson et al. 2007 USA	*N* = 20; 10 M, 10 W Recreationally active Age *(y)*: M: 24.5 ± 1.1; W: 25.4 ± 2.0 Weight *(kg):* M: 73.1 ± 2.4; W: 58.3 ± 1.9 BMI: M: 22.9 ± 1.6; W: 22.2 ± 0.4 VO_2_ max: M: 56.6 ± 2; W: 48.9 ± 2.6 ml/kg/min	Early follicular (blood levels measurements) Regular menstrual cycle reported (24–32 days)	NO	Three-day dietary record at the beginning, middle, and end of the study. Dietary energy intake on the day before test was individualized	Overnight fast and standardized breakfast of moderate/low glycemic index three hours before the test Cycling 90 min at 45% VO_2_ peak 60 min at 65% VO_2_ peak	6/9
Henderson et al. 2008 USA	*N* = 20; 10 M, 10 W Recreationally active Age *(y)*: M: 24.5 ± 1.1; W: 25.4 ± 2.0 Weight *(kg):* M: 73.1 ± 2.4; W: 58.3 ± 1.9 BMI: M: 22.9 ± 1.6; W: 22.2 ± 0.4 VO_2_ max: M: 56.6 ± 2; W: 48.9 ± 2.6 ml/kg/min	Early follicular (blood levels measurements) Regular menstrual cycle reported (24–32 days)	NO	Three-day dietary record at the beginning, middle, and end of the study. Dietary energy intake on the day before test was individualized	Overnight fast and standardized breakfast three hours before the test Cycling 90 min at 45% VO_2_ peak 60 min at 65% VO_2_ peak	5/9
Horton et al. 1998 USA^§^	*N* = 27; 14 M, 13 eumenorrheic W Sedentary: 6 M, 6 W Cyclists and triathlete: 8 M, 7 W Age *(y):* sedentary: M: 27 ± 3, W: 25 ± 3 Weight *(kg):* sedentary: M: 74.1 ± 6.7, W: 60.7 ± 6.2 BMI: not reported VO_2_ max: sedentary: M: 42.9 ± 3.7; W: 34.3 ± 3.8 ml/kg/min	Follicular (menstrual cycle history and blood levels measurements)	NO	Controlled diet for three days before each study day	10-h fast Cycling 120 min at 40% VO_2_ max	6/9
Keim et al. 1996 USA	*N* = 20; 10 M, 10 W Sedentary Age (y): M: 30 ± 1; W: 31 ± 1 Weight (kg): M: 79.2 ± 3.0; W: 53.1 ± 1.6 BMI: not reported VO_2_ max normalized to fat-free mass: M: 60.9 ± 4.55; W: 60.5 ± 4.41 ml/kg/min	Not reported	Not reported	Usual diet	Post-absorptive Cycling incremental test at 30, 40, 50, 60% VO_2_ max	6/9
Kuo et al. 2005 USA	*N* = 12; 6 M, 6 W Recreationally active Age *(y):* M: 21.2 ± 0.6; W: 22.8 ± 2.1 Weight *(kg):* M: 71.0 ± 4.8; W: 51.1 ± 1.4 BMI: Not reported VO_2_ max: M: 48.2 ± 4.2; W: 50.5 ± 1.9 ml/kg/min	Not reported	Not reported	Three-day dietary records were completed before each experimental trial	Same breakfast two hours before reporting to the laboratory for each trial Cycling exercise bouts, two exercise tasks 89 min at 45% VO_2_ peak 60 min at 65% VO_2_ peak	4/9
Lamont et al. 2001b^§^ USA	*N* = 14; 7 M, 7 W Sedentary: 2 M, 2 W Recreationally active: 2 M, 2 W Runners/triathletes: 3 M, 3 W Age *(y):* M: 30.71 ± 9.39; W: 30.57 ± 3.03 Weight *(kg):* M: 77.35 ± 3.35; W: 59.41 ± 2.98 BMI: not reported VO_2_ max: M: 46.2 ± 2.91; W: 42.2 ± 3.34 ml/kg/min	Follicular (n = 6) (ovulation kit)	Not reported	Dietitian designed a weekly meal plan for each subject	15 h post-absorptive Cycling 60 min at 50% VO_2_ max	5/9
McKenzie et al. 2000 Canada	*N* = 14; 6 M, 8 eumenorrheic W Sedentary Age *(y):* M: 26.9 ± 3.4; W: 23.7 ± 1.8 Weight *(kg):* M: 78.8 ± 12.1; W: 59.0 ± 9.0 BMI: not reported VO_2_ max: M: 45.9 ± 4.4; W: 37.7 ± 6.1 ml/kg/min	Mid follicular (method not specified)	YES (n = 3) NO (n = 5)	Four-day individual flesh-free, isoenergetic and isonitrogenous to their habitual diet dietary checklist and record. Pre-packaged diet on the day before, and the day of each exercise testing session	12-h fast Cycling 90 min at 60% VO_2_ peak	6/9
Mittendorferet al. 2002 USA	*N* = 10; 5 M, 5 premenopausal W Sedentary Age *(y):* M: 33 ± 3; W: 29 ± 4 Weight *(kg):* M: 78 ± 2; W: 57 ± 2 BMI: M: 25 ± 1; W: 21 ± 1 VO_2_ max: M: 37.0 ± 2; W: 35.0 ± 1 ml/kg/min	Follicular (method not specified)	Not reported	Not reported	At 19:00 day before trial standard meal, at 22:30 liquid formula Fast the day of the trial Cycling 90 min at 50% VO_2_ peak	5/9
Roepstorff et al. 2006 Denmark	*N* = 17; 8 M, 9 eumenorrheic W Recreationally active Age *(y):* M: 25 ± 1; W: 24 ± 1 Weight *(kg):* M: 79.5 ± 2.8; W: 65.0 ± 2.3 BMI: not reported VO_2_ max: M: 55.6 ± 1.2; W: 48.8 ± 1.3 ml/kg/min	Mid follicular (method not specified) Regular menstrual cycle reported (28–35 days)	NO	Eight days preceding the main trial, all subjects consumed an isoenergetic diet	Overnight fast Cycling 90 min at 60% VO_2_ peak	5/9
Ruby et al. 2002 USA^§^	*N* = 11; 5 M, 6 regularly menstruating W Sedentary: 1 M, 2 W Triathletes: 4 M, 4 W Age *(y):* M: 25.0 ± 2.0; W: 23.6 ± 1.1 Weight *(kg):* M: 68.2 ± 2.7; W: 60.1 ± 3.7 BMI: not reported VO_2_ max: M: 61.7 ± 1.3; W: 48.2 ± 1.1 ml/kg/min	Luteal and follicular (day of menses and morning oral temperature record and blood levels measurements) Regular menstrual cycle reported	NO	Two-day diary record before the submaximal test	10 h post-absorptive Cycling 25 min at 70% lactate threshold followed by 25 min at 90% lactate threshold	5/9
Steffensen et al. 2002 Denmark	*N* = 42; 21 M, 21 eumenorrheic W Sedentary: 7 M, 7 W Recreationally active: 7 M, 7 W Endurance trained:7 M, 7 W Age *(y)*: sedentary: M: 27 ± 2; W: 27 ± 1 recreationally active: M: 23 ± 1; W: 26 ± 1 Weight *(kg):* sedentary: M: 82.9 ± 5.7; W: 65.0 ± 2.8 recreationally active: M: 76.2 ± 1.9; W: 59.0 ± 2.5 BMI: not reported VO_2_ max: sedentary: M: 44.8 ± 2.9; W: 41.3 ± 0.8 ml/kg/min recreationally active: M: 55.0 ± 0.1; W: 50.7 ± 1.4 ml/kg/min	Mid follicular (blood levels measurements) Regular menstrual cycle reported (28–35 days)	NO	Five-day self-reported dietary record 8 days controlled, isoenergetic diet preceding the trial	Overnight fast Cycling 90 min at 60% VO_2_ peak	5/9
Tarnopolsky et al. 2007 Canada	*N* = 12; 5 M, 7 eumenorrheic W Recreationally active Age *(y):* M: 24.4 ± 3.8; W: 22.3 ± 1.4 Weight *(kg):* M: 79.9 ± 19.8; W: 65.2 ± 6.0 BMI: not reported VO_2_ max: M: 42.9 ± 7.3; W: 36.9 ± 6.6 ml/kg/min	Mid follicular (method not specified)	YES (n = 5)	Four-day dietary records one week before the start and completion of training	Formula supplement four hours before the start of exercise Cycling at 60% VO_2_ peak	6/9
Venables et al. 2005 UK	*N* = 300; 157 M, 143 W Recreationally active Age *(y):* M: 30 ± 11; W 32 ± 12 Weight *(kg):* M: 84.6 ± 14.8; W: 66.9 ± 11.1 BMI: M: 26 ± 4; W: 25 ± 4 VO_2_ max: M: 50.7 ± 0.9; W: 41.4 ± 0.9 ml/kg/min	Not reported	Not reported	Not reported	4-h fast Walking on treadmill Incremental exercise to exhaustion from 30 to 90% VO2 peak	7/9
White et al. 2003 USA	*N* = 18; 9 M, 9 premenopausal W Recreationally active Age *(y):* M: 27.4 ± 1.5; W: 27.2 ± 4.1 Weight *(kg):* M: 79.4 ± 2.7; W: 65.5 ± 3.3 BMI: Not reported VO_2_ max: M: 45.0 ± 1.6; W: 41.5 ± 2.8 ml/kg/min	Mid follicular (Menstrual cycle history) Normal cycle for previous 6 months	NO	Two-day dietary log to assess dietary habits Standard dietary instructions during the 3 days before the exercise trial	18-h fast Cycling 60 min at 65 ± 5% VO_2_ max	6/9

Data are presented as reported in the original full text. Study quality assessed by NIH Quality Assessment Tool for Before–After (Pre–Post) Studies. *BMI* body mass index; *M* men; *min* minute; *VO*_*2*_* max* maximum oxygen consumption; *VO*_*2*_* peak* peak oxygen uptake**; ***W* women; *y* years;

# data from the two individual studies by Friedlander et al. (1998; 1999) were merged

^§^ Excluded from the quantitative analysis (mixed sedentary subjects and athletes)

**Table 2:** Participant’s features, pre-testing and testing conditions and quality of studies carried out in healthy endurance trained athletes and included in the qualitative analyses (*N* = 17)

Study *Country*	Participants	Menstrual cycle phase	Oral contraception	Diet Assessment	Pre-testing condition and testing session	Study quality
Abramowicz et al. 2005 UK	*N* = 12; 6 M, 6 W Triathletes Age *(y):* M: 25 ± 6; W: 30 ± 5 Weight *(kg):* M: 74.7 ± 6.8; W: 62.8 ± 7.9 BMI: not reported VO_2_ max: M: 4.9 ± 0.77; W: 3.17 ± 0.4 L/min	Balance of follicular and luteal phase in trials (menstrual cycle history)	NO	Seven-day dietary record for habitual dietary intake; experimental diet throughout the duration of the study	3 h following ingestion of pre-exercise meal and final supplement Cycling 60 min at 60% VO_2_ max	6/9
Goedecke et al. 2000 South Africa	*N* = 61; 45 M, 16 W Cyclists Age *(y):* M: 32 ± 19; W: 29 ± 5 Weight *(kg):* M: 77.3 ± 9.3; W: 60.4 ± 5.3 BMI: not reported VO_2_ peak: M: 57.6 ± 6.7; W: 50.8 ± 6.3	Not reported	Not reported	Weighed dietary record 3 days before the experimental trial	12-h overnight fast Cycling steady-state exercise at 41%, 63%, and 80% VO_2_ peak	6/9
Horton et al. 2006 USA	*N* = 24; 13 M, 11 W Endurance trained Age *(y):* M: 33.8 ± 6.2; W: 34.0 ± 6.3 Weight *(kg):* M: 73.3 ± 7.5; W: 56.9 ± 7.7 BMI: M: 22.4 ± 1.5; W: 20.5 ± 1.6 VO_2_ max normalized to LBM: M: 65.1 ± 7.5; W: 64.4 ± 6.4 ml/kg/min	Mid luteal (blood levels measurements) Regular menstrual cycle (> 11 cycle over the past year)	NO	A controlled experimental diet for three days before the study day	Snack at 22:00 and fast until the end of test Cycling 90 min at 85% of each lactate threshold (~ 51% VO_2_ max)	7/9
Horton et al. 1998 USA^#^	*N* = 27; 14 M, 13 eumenorrheic W Sedentary: 6 M, 6 W Cyclists and triathlete: 8 M, 7 W Age *(y):* athletes: M: 25 ± 4; W: 27 ± 5 Weight *(kg):* athletes: M: 69.1 ± 7.0; W: 57.8 ± 6.5 BMI: not reported VO_2_ max: athletes: M: 64.4 ± 3.7; W: 55.3 ± 6.6 ml/kg/min	Follicular (menstrual cycle history and blood levels measurements)	NO	Controlled diet for three days before each study day	10-h fast Cycling 120 min at 40% VO_2_ max	6/9
Knechtle et al. 2004 Switzerland	*N* = 36; 19 M, 17 W Triathletes or cyclists Age *(y):* M: 34.1 ± 6.2; W: 32.1 ± 8.6 Weight *(kg):* M: 72.7 ± 5.8; W: 60.1 ± 4.1 BMI: not reported VO_2_ max: M: 61.4 ± 4.0; W: 52.8 ± 4 ml/kg/min	Not reported	YES = 4 NO = 13	High rich carbohydrate dinner the night before the test	Overnight fast Cycling or running 3 stages endurance test 30 min each endurance test + 15 min rest between each endurance test at 55%, 65%, 75% VO_2_ peak	5/9
Lamont et al. 2001a USA^#^	*N* = 14; 7 M, 7 W Runners/triathletes: 3 M, 3 W Moderately active: 2 M, 2 W Sedentary: 2 M, 2 W Age *(y):* M: 30.71 ± 9.39; W: 30.57 ± 3.03 Weight *(kg):* M: 77.35 ± 3.35; W: 59.41 ± 2.98 BMI: Not reported VO_2_ max: M: 46.2 ± 2.91; W: 42.2 ± 3.34 ml/kg/min	Follicular (n = 6) (ovulation kit)	Not reported	Dietician designed a weekly meal plan for each subject	15 h post-absorptive Cycling 60 min at 50% VO_2_ max	5/9
Phillips et al. 1993 Canada	*N* = 12; 6 M, 6 eumenorrheic W Runners Age *(y)*: M: 23.3 ± 3.9; W: 23.0 ± 4.9 Weight *(kg):* M: 64.1 ± 5.4; W: 58.1 ± 5.4 BMI: not reported VO_2_ max normalized to fat-free mass: M: 66.1 ± 7.6; W: 67.5 ± 5.4 ml/kg/min	Mid follicular (method not specified) Normal cycle length (27–33 days)	NO	Four-day food records collected immediately before the study Experimental diets: 2-day rotating menu for the entire 10-day adaptation, but fixed composition during the nitrogen balance period (3 days)	High-CHO breakfast 1-h prior test Treadmill 90 min at 65% VO_2_max	6/9
Powers et al. 1980 USA	*N* = 8; 4 M, 4 W Runners Age range *(y):* 22–35 Weight *(kg):* Not reported BMI: not reported VO_2_ peak: not reported	Not reported	Not reported	Not reported	12 h post-absorptive Treadmill 90 min at 65% VO_2_ max	6/9
Riddell et al. 2003 Canada	*N* = 14; 7 M, 7 eumenorrheic W Runners Age *(y):* M: 25.7 ± 4.6; W: 23.3 ± 1.5 Weight *(kg):* M: 77.6 ± 6.8; W: 61.5 ± 8.3 BMI: not reported VO_2_ max normalized to LBM: M: 68.9 ± 8.2; W: 65.7 ± 6.3 ml/kg/min	Mid follicular (method not specified)	Not reported	Four-day dietary records Same nutrient intake on the 2 days preceding the experimental trials	Snack formula 90 min prior start of the exercise 20 min prior and during exercise intake of either carbohydrate (8% solution) or artificially flavored placebo (aspartame flavored drink) Cycling 90 min at 60% VO_2_ peak	5/9
Roepstorff et al. 2002 Denmark	*N* = 14; 7 M, 7 eumenorrheic W Endurance trained Age *(y):* M: 26 ± 1; W: 25 ± 1 Weight *(kg):* M: 75.2 ± 1.8; W: 65.9 ± 3.3 BMI: Not reported VO_2_ max normalized to LBM: M: 71.7 ± 0.6; W: 71.0 ± 1.5 ml/kg/min	Mid follicular (method not specified) Cycle length between 28 and 35 days	NO	Five not consecutive days weighted food record Controlled, isocaloric diet eight days preceding the experiment	Overnight fast Cycling 90 min at 58% VO_2_ peak	5/9
Romijn et al. 2000 USA	*N* = 13; 5 M, 8 eumenorrheic W Cyclists Age *(y):* M: 24 ± 2; W: 27 ± 1 Weight *(kg):* M: 75.2 ± 3.6; W: 60.6 ± 3.2 BMI: Not reported VO_2_ max normalized to LBM: M: 73.6 ± 3.5; W: 70.1 ± 2.0 ml/kg/min	Not reported	Not reported	Weight-maintaining diet containing at least 300–400 g of carbohydrates/die	12 h post-absorptive Cycling 60 min at 65% VO_2_ max Evaluation at 25%, 65%, 85% VO_2_ max after 20–30 min	5/9
Ruby et al. 2002^#^ USA	*N* = 11; 5 M, 6 regularly menstruating W Triathletes 4 M, 4 W Sedentary 1 M, 2 W Age *(y):* M: 25.0 ± 2.0; W: 23.6 ± 1.1 Weight *(kg):* M: 68.2 ± 2.7; W: 60.1 ± 3.7 BMI: not reported VO_2_ max normalized to fat-free mass: M: 67.4 ± 1.3; W: 56.5 ± 1.4 ml/kg/min	Luteal and follicular (Day of menses and morning oral temperature record and blood levels measurements) Reported regular menstrual flow	NO	Two-day diary record before the submaximal test	10 h post-absorptive Cycling 25 min at 70% lactate threshold followed by 25 min at 90% lactate threshold	5/9
Steffensen et al. 2002 Denmark	*N* = 42; 21 M, 21 eumenorrheic W Endurance trained: 7 M, 7 W Sedentary: 7 M, 7 W Recreationally active: 7 M, 7 W Age *(y)*: endurance trained: M: 26 ± 1; W: 25 ± 1 Weight *(kg):* endurance trained: M: 75.2 ± 1.8; W: 65.9 ± 3.3 BMI: not reported VO_2_ max: endurance trained: M: 63.3 ± 0.8; W: 58.1 ± 1.3 ml/kg/min	Mid follicular (blood levels measurements) Normal cycle length of 28–35 days	NO	Five-day self-reported dietary record 8 days controlled, isoenergetic diet preceding the trial	Overnight fast Cycling 90 min at 60% VO_2_ peak	5/9
Tarnopolsky et al. 1990 Canada	*N* = 12; 6 M, 6 eumenorrheic W Runners Age *(y):* M: 20 ± 0.6; W: 21.5 ± 0.8 Weight *(kg):* M: 66.9 ± 2.1; W: 58.4 ± 2.2 BMI: not reported VO_2_ max normalized to LBM: M: 74.9 ± 0.9; W: 74.7 ± 1.7 ml/kg/min	Mid follicular (method not specified) Normal cycle length of 28–34 days	NO	Detailed food records 2 weeks before the testing session For 2 days before and on the day of test isocaloric pre-packaged caffeine-free diet	11 h post-absorptive Treadmill 90–101 min, 15.5 km at 65% VO_2_ max	5/9
Tarnopolsky et al. 1997 Canada	*N* = 16; 8 M, 8 eumenorrheic W Runners Age *(y):* M: 22.1 ± 2.2; W: 20.3 ± 0.89 Weight *(kg):* M: 72.9 ± 5.4; W: 61.1 ± 8.5 BMI: Not reported VO_2_ max normalized to LBM: M: 63.8 ± 2.6; W: 65.1 ± 3.5 ml/kg/min	Mid follicular (method not specified)	YES (n = 3)	Four-day diet records Individual designed isoenergetic and isonitrogenous diets for the three trials	Fasted state Cycling 90 min at 65% VO_2_ peak Post-exercise supplements (three different conditions)	7/9
Wallis et al. 2006 UK	*N* = 16; 8 M, 8 eumenorrheic W Endurance trained Age *(y):* M: 32 ± 2; W: 32 ± 3 Weight *(kg):* M: 78.3 ± 2.6; W: 65.2 ± 2.2 BMI: not reported VO_2_ max normalized to LBM: M: 61.4 ± 1.5; W: 63.6 ± 2.4 ml/kg/min	Follicular (blood levels measurements) Normal menstrual cycle length of 25–32 days	NO	Specific exercise–diet regimen in the four 7 days leading up to the experimental trials Provided diet the day before the experimental trial	Overnight fast (> 10 h) At start and during exercise intake of either carbohydrate (10.9% glucose solution) or plain water (placebo) Cycling 120 min at 67% VO_2_ max	5/9
Zehnder et al. 2005 Switzerland	*N* = 18; 9 M, 9 eumenorrheic W Cyclists or triathletes Age *(y):* M: 34 ± 4; W: 30 ± 4 Weight *(kg):* M: 73.9 ± 8.4; W: 58.9 ± 5.6 BMI: Not reported VO_2_ max normalized to LBM: M: 65.0 ± 7.0; W: 53.0 ± 4.0 ml/kg/min	Mid follicular (method not specified)	Not reported	Two days before the trials, diet control and nutrition protocol for each meal Consumption of carbohydrate-rich meals day before exercise test	Overnight fast Cycling 120 min at 60–65% VO_2_ peak	5/9

Data are presented as reported in the original full text. Study quality assessed by NIH Quality Assessment Tool for Before–After (Pre–Post) Studies. Abbreviations: *BMI* body mass index; *LBM* lean body mass; *M* men; *min* minute; *VO*_*2*_* max* maximum oxygen consumption; *VO*_*2*_* peak* peak oxygen uptake; *W peak* peak power output; *W* women; *y* years

^#^ Not included in the quantitative analysis

**Table 3:** Main outcomes, findings and suggested mechanisms for sex-based differences of studies carried out in sedentary or recreationally active healthy subjects and included in the qualitative analyses (*N* = 28)

Study *Country*	Sample type	Main outcome measures	Main findings	Suggested mechanisms for the sex-based differences in substrate utilization
Arner et al. 1990 Sweden	Microdialysis, blood	Glycerol level in the abdominal and gluteal subcutaneous adipose tissue Plasma glycerol	Glycerol level in the abdominal region during exercise: W > M* Plasma glycerol: W > M**	Fat Different pattern of adrenergic activation of lipolysis Sex hormones
Blatchford et al. 1985 USA	Blood	RER Plasma FFA Plasma glycerol Plasma lactate % Fat metabolism	RER: M > W* at 15, 45, 90 min of exercise Plasma FFA: W > M* at 45 and 90 min of exercise Plasma Glycerol: W > M* at 45 min of exercise	Fat Sex hormones Different pattern of adrenergic activation of lipolysis
Boschmann et al. 2002 USA	Microdialysis, blood	Dialyzed glycerol concentration abdominal, femoral adipose tissue and muscle Dialyzed lactate concentration in abdominal, femoral adipose tissue and muscle Dialyzed citrate concentration abdominal, femoral adipose tissue and muscle Respiratory quotient	Dialysed glycerol in muscle: W > M** at 60 min of exercise	Fat Different pattern of adrenergic activation of lipolysis Intramuscular lipid content (W > M)
Burguera et al. 2000 USA	Blood, breath	Plasma glucose Plasma palmitate Plasma lactate Systemic palmitate rate of appearance Leg palmitate release Leg palmitate uptake	No sex difference	Fat: No sex differences observed
Carter et al. 2001 Canada	Blood, breath	VO_2_ peak Hearth rate RER CHO oxidation Fat oxidation Glucose rate of appearance Glucose rate of disappearance Glucose MRC Plasma lactate Plasma glucose Glycerol rate of appearance Glycerol rate of disappearance Plasma glycerol Plasma FFA	VO_2_ peak: W < M*** RER: W < M*** (pre–post training) CHO oxidation: W < M** Fat oxidation: M < W*** Glucose rate of appearance and rate of disappearance: no sex difference Glucose MCR: W < M* at 75 min and 90 min Plasma Lactate and Glucose: no sex difference Glycerol rate of appearance and glycerol rate of disappearance: W > M** Plasma glycerol: no sex difference Plasma FFA: W > M*	Fat and carbohydrates Sex hormones
Cheneviere et al. 2011 Switzerland	Breath	RER Fat oxidation rate CHO oxidation rate CHO oxidation %EE Lipid oxidation %EE MFO	RER: M > W* from 35 to 85% VO_2_ max Fat oxidation rate: W > M* from 35 to 85% VO_2_ max MFO: W > M** from 35 to 85% VO_2_ max	Fat Body composition (body fat: W > M, fat-free mass: W < M) Muscle fiber distribution (type I: W > M) Different pattern of adrenergic activation of lipolysis
Cunningham et al. 1990 USA	Breath	VO_2_ peak RER Heart rate	RER: no sex difference	No sex differences observed
Dasilva et al. 2011 Brazil	Breath	Fat oxidation CHO oxidation Contribution of fat and CHO to EE MFO Fat_max_ Fat_min_ Fat_max_ zone VO_2_ VCO_2_ Heart rate, % heart rate max RER EE exercise	MFO: no sex differences Fat_max_: W > M** Fat_min_: W > M*** Fat_max_ zone W > M* CHO oxidation: M > W* EE exercise: M > W* Contribution of fat to EE: W > M* Contribution of CHO to EE: M > W** Absolute CHO oxidation rate: M > W*** Absolute fat oxidation rate: no sex differences VO_2_: M > W* Heath rate, % heart rate max: no sex differences	Fat and carbohydrates Sex hormones Different pattern of adrenergic activation Different enzymatic activity Muscle fiber distribution (type I: W > M)
Davis et al. 2000 USA	Blood and breath	Plasma glucose Plasma lactate Plasma glycerol Plasma NEFA Plasma β-hydroxybutyrate Glucose rate of disposal CHO oxidation Lipid oxidation	Plasma glucose: no sex difference Plasma glycerol: W > M** during exercise Plasma NEFA: W > M** during exercise Plasma β-hydroxybutyrate: W > M** during exercise CHO oxidation: M > W* Lipid oxidation: no sex difference	Fat Different pattern of adrenergic activation Body composition (body fat: W > M, fat-free mass: W < M)
Devries et al. 2007 Canada	Muscle, breath	CHO oxidation Fat/lipid oxidation IMCL mean size IMCL/μm2 IMCL area density IMCL-t mitochondria IMCL net use VO_2_ peak RER	CHO oxidation: M > W** CHO oxidation: < in both sexes*** comparing 60–90 min with 30 min Fat oxidation: W > M* Fat oxidation: > in both sexes*** comparing 60–90 min with 30 min CHO Ox/Fat Ox: M > W* IMCL/μm2: W > M** IMCL area density: W > M* IMCL-touching mitochondria: W > M* post-exercise IMCL net use: no sex differences VO_2_: M > W* VO_2_ to FFM: no sex differences RER—rest: no sex differences RER—exercise: M > W* RER: < in both sexes*** comparing 60–90 min with 30 min	Fat Sex hormones mRNA expression of genes associated with free fatty acid transport to plasma and mitochondrial membranes during exercise (W > M) Carbohydrates Sex hormones
Devries et al. 2006 Canada	Muscle, blood and breath	RER Plasma glucose Plasma lactate Glucose rate of appearance, rate of disappearance, MCR Muscle glycogen (PG and MG) utilization Contribution of plasma glucose and muscle glycogen to CHO oxidation	RER: FP < M* during exercise; LP < M* at 75’, 90’ Plasma glucose and Lactate: no sex difference Glucose rate of appearance: FP and LP < M* Glucose rate of disappearance: FP and LP < M* Glucose MCR: FP and LP < M* and ** Muscle PG utilization: LP < M* Muscle glycogen contribution to CHO oxidation: FP > M* Plasma Glucose contribution to CHO oxidation: FP < M*	Carbohydrates Sex hormones
Friedlander et al. 1998 USA ^#^	Blood and breath	VO_2_ peak Hearth rate RER Plasma glucose Plasma lactate Glucose rate of appearance, rate of disappearance and MCR Glucose rate of oxidation Oxidative energy source Glucose recycling rate	RER: W < M* (post-training) Glucose recycling rate: W < M* (pre- and post-training) Glucose rate of oxidation: W < M* pre-training %EE CHO oxidation: W < M*post-training Plasma Lactate: W < M* post-training	Carbohydrates Sex hormones Muscle glycogen concentration (M > W) Receptor availability and affinity to hormone levels Differences in glucose recycling Fat Sex hormones
Friedlander et al. 1999 USA ^#^	Blood and breath	VO_2_ peak Hearth rate RER Plasma glucose Plasma FFA Plasma glycerol Palmitate and glycerol rate of appearance, rate of disappearance and MCR Glycerol flux rates Palmitate rate of oxidation Rate total FFA oxidation	Total fat oxidation rate: W > M* post-training exercise RER: M > W* post-training exercise Glycerol rate of appearance: W > M* pre- and post-training exercise	Fat Sex and adrenergic hormones’ interaction
Hellström et al. 1996 Sweden	Microdialysis technique, blood	Plasma glycerol Serum FFA Glycerol levels in dialysate of AT from abdominal region Dialysate lactate	Plasma glycerol: W > M*** Serum FFA: W > M** Glycerol levels in dialysate of AT from abdominal region: W > M** *P* value from the graphs. Results from the control condition	Fat Body composition Different pattern of adrenergic activation of lipolysis
Henderson et al. 2007 USA	Blood and breath	Exercise EE VO_2_ peak RER Plasma glycerol Plasma FA Glycerol rate of appearance FA rate of appearance Ratio of FA rate of appearance and glycerol rate of appearance % of FA disposal oxidized Lipid oxidation % EE CHO oxidation % EE fat oxidation	RER: M > W* at 45% and 65% VO_2_ peak Glycerol rate of appearance: W > M* at 65% VO_2_ peak % EE CHO oxidation: M > W* at 45% and 65% VO_2_ peak % EE fat oxidation: W > M* at 45% and 65% VO_2_ peak	Fat Body composition (body fat: W > M, fat-free mass: W < M)
Henderson et al. 2008 USA	Blood and breath	Exercise EE VO_2_ peak Plasma glucose Plasma lactate Glucose rate of appearance Glucose rate of disappearance Glucose MCR	Blood glucose: no sex difference Blood lactate: M > W* during exercise at 45% VO_2_ peak Glucose rate of appearance and glucose rate of disappearance: no sex difference Glucose MCR: M > W* during exercise at 45% VO_2_ peak	Carbohydrates Different patterns of glycemia maintenance
Horton et al. 1998^§^ USA	Blood and breath	RER CHO oxidation Fat oxidation Protein oxidation % EE CHO oxidation % EE fat oxidation % EE protein oxidation Plasma FFA Plasma glucose Plasma glycerol Plasma β-hydroxy-butirric acid Plasma lactate	RER: M > W* CHO oxidation: M > W*** Fat oxidation: no sex difference Protein oxidation: M > W** %EE CHO oxidation: M > W** %EE Fat oxidation: W > M* %EE protein oxidation: no sex difference Plasma FFA: W > M** N.B. Results reported by sex, regardless the level of physical activity (trained or untrained)	Carbohydrates Sex-based differences in maintenance of glycemia Different enzymatic activity Sex hormones Fat Different pattern of adrenergic activation Sex hormones Cortisol Proteins Sex-based differences not discussed
Keim et al. 1996 USA	Breath	RER CHO oxidation Fat oxidation	CHO oxidation: M > W* at 30% VO_2_ max Fat oxidation: M < W* at 30% VO_2_ max NB. A comparison to test for sex effect was done with a different set of men and women who were matched by body fat percentage	No sex differences observed
Kuo et al. 2005 USA	Breath	VO_2_ VCO_2_ RER % energy from CHO % energy from lipid Energy from CHO oxidation Energy from lipid oxidation EE	RER – during exercise: no significant sex differences RER – post-exercise: no sex differences Relative Substrate oxidation: no significant sex differences	No sex differences observed
Lamont et al. 2001b^§^ USA	Blood and breath	Leucine rate of appearance Lysine rate of appearance Leucine oxidation NOLD Plasma urea nitrogen Plasma FFA Plasma glucose Non protein RER % CHO % fat % protein	Leucine and lysine rate of appearance: no sex differences Leucine oxidation—exercise: M > W* Leucine oxidation—rest or recovery: no sex differences NOLD– exercise: W > M* NOLD – rest: no sex differences %CHO: M > W* %Fat: W > M* %Protein: M > W* Plasma urea nitrogen or FFA: no sex differences Plasma glucose at 15 min: M > W* Non protein RER: M > W***	Proteins Different enzymatic activity Fat and carbohydrates Different pattern of adrenergic activation
McKenzie et al. 2000 Canada	Muscle, blood, breath	VO_2_ peak RER CHO oxidation Fat oxidation Leucine oxidation Leucine Flux NOLD BCOAD Urea nitrogen excretion Creatinine excretion Plasma lactate Plasma glucose Muscle glycogen	RER: M > W* CHO oxidation: M > W* (pre- and post-training) Fat oxidation: W > M* (pre- and post-training) Leucine oxidation: M > W** (pre- and post-training) Leucine Flux: W < M* (at all time points) BCOAD: decreased post-training, no sex difference Urea Nitrogen excretion: M > W * Creatinine excretion: M > W** Plasma glucose, plasma lactate and muscle glycogen: no sex difference	Proteins Different enzymatic activity Carbohydrates Difference in hepatic glycogen sparing (> in women) Fat Not explained
Mittendorfer al. 2002 USA	Blood and Breath	RER Fat oxidation CHO oxidation Glycerol rate of appearance Palmitate rate of appearance and rate of disappearance Rate of total plasma FFA oxidation Rate of non-plasma fatty acids oxidation	RER: no sex difference Fat oxidation: no sex difference Glycerol rate of appearance: W > M* Palmitate rate of appearance and rate of disappearance: W > M* Rate of tot plasma FFA oxidation: W > M* Rate of non-plasma fatty acids oxidation: M > W*	Fat Different pattern of adrenergic activation Body composition
Roepstorff et al. 2006 Denmark	Muscle, blood and breath	Fat oxidation rate Blood glucose Blood lactate Muscle glycogen Muscle lactate Creatine Phosphocreatine RER VO_2_ α1AMPK, α2AMPK, ACCβ, AMPK activity ATP, ADP	Fat oxidation: W > M* at 30, 45,60,75 and 90 min RER: M > W* at 60 and 90 min VO_2_: M > W*** Blood glucose: M > W* Creatine: M > W* α1AMPK, α2AMPK, ACCβ, AMPK activity and ATP, ADP: no significant sex difference	Fat Muscle fiber distribution (type I: W > M) Muscle capillarization (W > M)
Ruby et al. 2002^§^ USA	Blood and breath	Glucose rate of appearance and rate of disposal Plasma lactate Plasma glycerol Muscle glycogen to total CHO oxidation Insulin CHO oxidation Fat oxidation % Fat % CHO RER VO_2_ Kcal/min (TEE)	Glucose rate of appearance to FFM at 70% and 90% lactate threshold: no sex differences Glucose rate of appearance to body mass at 90% lactate threshold: significant M > W *(not reported p value, M* = *36.4* ± *3.7, W* = *28.9* ± *4.8)* Glucose rate of disposal to body mass at 70% lactate threshold: no sex differences Glucose rate of disposal to body mass at 90% lactate threshold: significant M > W *(not reported p value, M* = *34.7* ± *3.4, W* = *28.4* ± *4.8)* Glucose concentration: W > M* at 70% lactate threshold Plasma glucose relative contributions to total CHO oxidation: W > M* at 70% and 90% lactate threshold Muscle glycogen relative contributions to total CHO: M > W* at 70% and 90% lactate threshold Fat oxidation: M > W* at 70% and 90% lactate threshold CHO oxidation: M > W* at 70% and 90% lactate threshold RER: no sex differences Kcal/min (TEE): M > W* at 70% and 90% lactate threshold	Carbohydrates Sex hormones Sex-based differences in maintenance of glycemia
Steffensen et al. 2002 Denmark	Muscle, blood and breath	RER Muscle MCTG	RER: no sex difference Muscle MCTG content: W > M*** Muscle MCTG usage during exercise: W > M***	Fat Muscle fiber distribution (type I: W > M) Different pattern of adrenergic activation Hormone-sensitive lipase
Tarnopolsky et al. 2007 Canada	Muscle, blood and breath	Plasma glucose Plasma Lactate Plasma FFA Plasma glycerol Plasma total triglyceride Insulin Citrate synthase enzyme (CS) SCHAD IMCL individual area IMCL area IMCL/μm^2^ IMCL-t mitochondria CHO oxidation Fat oxidation RER Heart rate VO_2_ peak Mitochondrial area Mitochondria/μm^2^ Individual mitochondria	Glycerol: W > M* FFA: W > M*** Insulin, triglycerides, glucose: no sex differences CS: both sex increase M > W* (M = 26%, W = 3%) SCHAD: both sex increase M > W** (M = 39%, W = 13%;) IMCL individual area: W > M* for pre-training IMCL/μm^2^: W > M** IMCL area: W > M* CHO oxidation: M > W* Fat oxidation: W > M* RER: M > W** sex effect VO_2_: M > W* sex effect VO_2_ to FFM: no significant sex effect	Fat Sex hormones Muscle lipid content (W > M)
Venables et al. 2005 UK	Breath	MFO Fat_max_ VO_2_ VCO_2_ RER Absolute fat oxidation Absolute CHO oxidation % fat oxidation % CHO oxidation	Absolute CHO oxidation—41–61% VO_2_ max: M > W** MFO per FFM kg—41–61% VO_2_ max: W > M** %Fat oxidation—41–61% VO_2_ max: W > M**	Fat Sex hormones Different adrenergic activation of lipolysis Muscle fiber distribution (type I: W > M)
White et al. 2003 USA	Blood and breath	Plasma FFA Plasma glycerol Plasma triglyceride Blood lactate CHO IMCL Heart rate RER	Lipid oxidation (Kcal FFM min): no sex differences IMCL: no sex differences	No sex differences observed

*AT* adipose tissue; *BCOAD* branched-chain 2-oxoacid dehydrogenase; *CHO* carbohydrate. *EE* energy expenditure; *FA* fatty acids; *Fat*_*max*_* zone* range of exercise intensities with fat oxidation rates within the 10% of fat oxidation rate at Fat_max_; *Fat*_*max*_ exercise intensity at which fat oxidation is maximal; *Fat*_*min*_ exercise intensity at which fat oxidation is minimal; *FFA* free fatty acid; *FFM* fat-free mass; *FP* follicular phase; *IMCL* intramyocellular lipid; *LP* luteal phase; *M* men; *MCTG* myocellular triacylglycerol; *MFO* maximal fat oxidation; *MG* macroglycogen; *min* minute; *NEFA* non esterified fatty acids; *NOLD* non-oxidative leucine disposal; *PG* proglycogen; *RER* respiratory exchange ratio; *SCHAD* short-chain-hydroxyacyl-CoA dehydrogenase; *TEE* total energy expenditure; *VCO*_*2*_ carbon dioxide production; *VO*_*2*_* max* maximum oxygen consumption; *VO*_*2*_* peak* peak oxygen uptake; *VO*_*2*_ oxygen uptake; *W* women

^*^Significant for *p* < 0.05; **significant for *p* < 0.01; ***significant for *p* < 0.001;

^#^ data from the two individual studies by Friedlander et al. (1998, 1999) were merged.

^§^ Excluded from the quantitative analysis (mixed sedentary subjects and athletes)

**Table 4:** Main outcomes, findings, and suggested mechanisms for sex-based differences of studies carried out in carried out in healthy endurance-trained athletes and included in the qualitative analyses (N = 17)

Study *Country*	Sample type	Main outcome measures	Main findings	Suggested mechanisms for the sex-based differences in substrate utilization
Abramowicz et al. 2005 UK	Blood and breath	RER CHO oxidation Fat oxidation Blood Lactate Plasma NEFA Plasma glycerol VO_2_	No significant differences	No sex-based difference observed
Goedecke et al. 2000 South Africa	Muscle, blood and breath	RER	RER: no sex difference	No sex-based differences observed
Horton et al. 2006 USA	Blood and breath	RER Non-protein RER CHO oxidation Protein oxidation Fat oxidation Glucose rate of appearance Glucose rate of disappearance Blood glucose oxidation Blood glycogen oxidation Blood lactate	RER and non-protein RER: no sex differences CHO oxidation (absolute rate): W < M* Protein oxidation (absolute rate): W < M*** Glucose rate of appearance (per KgBW): W < M**; (*per* KgLBM): W < M* Glucose rate of disappearance (*per* KgBW): W < M*; (*per* KgLBM): W < M (p = 0.065) Blood glucose oxidation (absolute): W < M*** Glycogen oxidation (absolute): W < M**; (*per* LBM) W < M*; (leg lean mass) W < M* Blood lactate: W < M*	Carbohydrates Different pattern of adrenergic activation Sex hormones
Horton et al. 1998^#^ USA	Blood and breath	RER CHO oxidation Fat oxidation Protein oxidation %EE CHO oxidation %EE fat oxidation %EE protein oxidation Plasma FFA Plasma glucose Plasma glycerol Plasma β-hydroxy-butirric acid Plasma lactate	RER: M > W* CHO oxidation: M > W*** Fat oxidation: no sex difference Protein oxidation: M > W** % EE CHO oxidation: M > W** % EE Fat oxidation: W > M* % EE protein oxidation: no sex difference Plasma FFA: W > M** N.B. Results reported by gender, regardless the level of physical activity (trained or untrained)	Carbohydrates Sex-based differences in glycemic maintenance Different enzymatic activity Sex hormones Fat Different pattern of adrenergic activation Sex hormones Cortisol Protein Sex-based differences not discussed
Knechtle et al. 2004 Switzerland	Blood and breath	Blood lactate Fat oxidation rate CHO oxidation rate EE% CHO oxidation EE% Fat oxidation RER	CHO oxidation rate: M > W* at all intensities % EE Fat oxidation: W > M* RER: W < M* at 65% and 75% VO_2_ peak	Fat Muscle lipid content (W > M) Sex hormones Muscle fiber distribution (type I: W > M)
Lamont et al. 2001a^#^ USA	Blood and breath	Leucine rate of appearance Leucine oxidation NOLD Lysine rate of appearance Plasma urea nitrogen Plasma FFA Plasma glucose Non-protein RER % fat % CHO % protein	Non-protein RER: W < M*** % fat: W > M* % CHO: M > W* % protein: M > W* Plasma glucose: M > W* Leucine rate of appearance: no sex difference Lysine rate of appearance: no sex difference Leucine oxidation: M > W* NOLD: W > M*	Proteins Different enzymatic activity Fat and carbohydrates Different pattern of adrenergic activation
Phillips et al. 1993 Canada	Blood and breath	Non-protein RER Lipid utilization CHO utilization Lipid/CHO ratio Protein utilization Protein contribution to %EE Plasma lactate Urea nitrogen excretion Leucine oxidation Leucine flux NOLD	Non-protein RER: M > W* Lipid utilization: no sex difference CHO utilization: M > W** Lipid/CHO ratio: W > M* Protein utilization: M > W* Protein contribution to % EE: M > W* Leucine oxidation: M > W** NOLD: no sex difference	Fat No sex-based differences observed Carbohydrates Different enzymatic activity Proteins No explanation for the higher absolute leucine oxidation in the males than in the females
Powers et al. 1980 USA	Blood and breath	% EE Fat oxidation RER Blood lactate	% EE Fat oxidation: no sex difference RER: no sex difference Blood lactate: no sex difference	No sex-based differences observed
Riddell et al. 2003 Canada	Blood and breath	Plasma glucose Plasma lactate Protein oxidation (urea concentration in urine) CHO oxidation endogenous CHO oxidation exogenous Fat oxidation RER	Fat oxidation: W > M* at 30 min NB: main finding only for placebo condition	Carbohydrates Sex hormones Different enzymatic activity
Roepstorff et al. 2002 Denmark	Muscle, blood and breath	Blood glucose Blood FA Blood glycerol Blood lactate Glucose rate of appearance Glucose rate of disappearance Plasma FA rate of appearance Plasma FA rate of disappearance Plasma Fat oxidation Plasma FA release Plasma FA tot uptake Muscle glycogen utilization MCTG RER Leg substrate utilization (% of total O_2_ uptake)	Glucose rate of appearance and rate of disappearance: W < M* Plasma FA release: W > M** MCTG usage during exercise: W > M Plasma FA: W > M* MCTG: W > M*	Fat Muscle lipid content
Romijn et al. 2000 USA	Blood and breath	Plasma glucose FFA uptake FA oxidation Glucose rate of disappearance Carbohydrate oxidation RER	No sex differences at 65% VO_2_ max Glucose rate of disappearance: M > W** at 25% VO_2_ max CHO oxidation: W > M** at 25% VO_2_ max	No sex-based differences observed
Ruby et al. 2002^#^ USA	Blood and breath	Glucose rate of appearance and rate of disposal Plasma lactate Plasma glycerol Muscle glycogen to total CHO oxidation Insulin CHO oxidation Fat oxidation % Fat % CHO RER VO_2_ TEE	Glucose rate of appearance to free-fat mass: no sex differences at 70% and 90% lactate threshold Glucose rate of appearance to body mass: significant M > W at 90% lactate threshold *(not reported p value, M* = *36.4* ± *3.7, W* = *28.9* ± *4.8)* Glucose rate of disposal to body mass: no sex differences at 70% lactate threshold Glucose rate of disposal to body mass: significant M > W at 90% lactate threshold *(not reported p value, M* = *34.7* ± *3.4, W* = *28.4* ± *4.8)* Glucose concentration: W > M* at 70% lactate threshold Plasma glucose relative contributions to total CHO oxidation: W > M* at 70% and 90% lactate threshold Muscle glycogen relative contributions to total CHO: M > W* at 70% and 90% lactate threshold Fat oxidation: M > W* at 70% and 90% lactate threshold CHO oxidation: M > W* at 70% and 90% lactate threshold RER: no sex differences TEE: M > W* at 70% and 90% lactate threshold	Carbohydrates Sex hormones Sex-based differences in glycemic maintenance
Steffensen et al. 2002 Denmark	Muscle, blood and breath	RER Muscle MCTG	RER: no sex difference Muscle MCTG content: W > M*** Muscle MCTG usage during exercise: W > M***	Fat Muscle fiber distribution (type I: W > M) Different pattern of adrenergic activation
Tarnopolsky et al. 1990 Canada	Muscle, blood and breath	Blood FFA Plasma urea nitrogen Plasma glycerol Plasma glucose Plasma lactate Muscle glycogen Fat utilization CHO utilization RER	RER: W < M** Fat utilization: W > M** CHO utilization: W < M** Plasma glucose: W > M* Plasma urea nitrogen: M > W*	Fat and carbohydrates Muscle fiber distribution (type I: W > M) Insulin and epinephrine
Tarnopolsky et al. 1997 Canada	Muscle, blood and breath	RER Plasma glucose Muscle glycogen	RER: M > W** during exercise	Sex-based differences not discussed
Wallis et al. 2006 UK	Blood and breath	Plasma glucose Plasma lactate Plasma FFA Plasma glycerol Glucose rate of appearance Glucose rate of disappearance MCR glucose Glycerol rate of appearance Glycerol rate of disappearance Muscle glycogen oxidation Fat oxidation CHO oxidation RER	Plasma FFA: W > M* Plasma glycerol: W > M* CHO endo oxidation rate: W < M* CHO endo oxidation %EE: W < M* NB: main finding only for placebo condition	Sex-based differences discussed only for supplementation groups
Zehnder et al. 2005 Switzerland	Muscle (magnetic resonance spectroscopy) blood and breath	VO_2_ peak Plasma lactate Plasma glucose Fat oxidation rate CHO oxidation rate Muscle glycogen IMCL reduction RER	IMCL reduction: M > W*** VO_2_ peak: M > W** (both not normalized and normalized to LBM) CHO oxidation rate: M > W* during all trial, M > W* at 2 h, M > W** at 3 h	Fat Different muscle lipid content (M > W) Different pattern of adrenergic activation Hormone-sensitive lipase

*CHO* carbohydrate. *EE* energy expenditure; *F* fatty acids; *FFA* free fatty acid; ***h*** hour; *IMCL* intramyocellular lipid; *M* men; *MCTG* myocellular triacylglycerol; *min* minute; *NEFA* non esterified fatty acids; *NOLD* non-oxidative leucine disposal; *RER* respiratory exchange ratio; *TEE* total energy expenditure; *VO*_*2*_* peak* peak oxygen uptake; *VO*_*2*_ oxygen uptake; *W* women.

*Significant for *p* < 0.05

**Significant for *p* < 0.01

***Significant for *p* < 0.001

^#^ Excluded from the quantitative analysis

### Quality of the included studies

Tables 1 and 2 also report the results of the analysis of the methodological quality and risk of bias for the included studies, as assessed by the NIH Study Quality Assessment Tools. Three items proved not applicable to the design of the studies considered for the present study (item 9: losses to follow-up after baseline; item 11: multiple assessments before and after the intervention; item 12: use of individual-level data); therefore, the score was calculated out of 9 rather than 12 items. The mean score of the 28 studies on sedentary/recreationally active subjects was 5.5 ± 0.64 (95% CI: 5.29 to 5.79; median: 5.5). The 17 studies on athletes/highly trained subjects had an average score of 5.53 ± 0.72 (95% CI: 5.16 to 5.90; median: 5.0). The Mann–Whitney *U* test revealed no significant difference between the scores of the two study groups (*p* = 0.81). In both cases, the most frequently unsatisfied criteria were items 3 (12 out of 45 studies; “*Were the participants in the study representative of those who would be eligible for the test/service/intervention in the general or clinical population of interest?*”), 4 (8 out of 45 studies; “*Were all eligible participants that met the pre-specified entry criteria enrolled?*”), 5 (12 out of 45 studies; “*Was the sample size sufficiently large to provide confidence in the findings?*”), and 8 (12 out of 45 studies; “*Were the people assessing the outcomes blinded to the participants' exposures/interventions?*”).

### Quantitative analysis

Of the 28 studies involving sedentary/recreationally active subjects and deemed eligible for the qualitative analysis, 21 contributed data to at least one of the planned meta-analyses (Blatchford et al. 1985; Burguera et al. 2000; Carter et al. 2001; Cheneviere et al. 2011; Dasilva et al. 2011; Davis et al. 2000; Devries et al. 2006; 2007; Friedlander et al. 1998, 1999; Henderson et al. 2007; 2008; Keim et al. 1996; Kuo et al. 2005; McKenzie et al. 2000; Mittendorfer et al. 2002; Roepstorff et al. 2006; Steffensen et al. 2002; Tarnopolsky et al. 2007; Venables et al. 2005; White et al. 2003).

Of the 17 studies conducted in athletic populations and deemed eligible for the qualitative analysis, 14 contributed data to at least one of the planned meta-analyses (Abramowicz and Galloway 2005; Goedecke et al. 2000; Horton et al. 2006; Knechtle et al. 2004; Phillips et al. 1993; Powers et al. 1980; Riddell et al. 2003; Roepstorff et al. 2002; Romijn et al. 2000; Steffensen et al. 2002; Tarnopolsky et al. 1990; 1997; Wallis et al. 2006; Zehnder et al. 2005).

Reasons for exclusion from the meta-analyses ranged from ‘mixed population’ (i.e., enrollment of recreationally active and athletes, without reporting data separately) to presence of sex imbalance (e.g., enrollment of more males than females), as detailed in Fig. 1. Regarding the presence of publication bias in the included studies, for those meta-analyses consisting of at least ten studies, the visual inspection of the funnel plots revealed no asymmetry for all the outcomes considered (VO_2_ peak by body weight and by lean body mass in sedentary subjects; VO_2_ peak by lean body mass in athletes; carbohydrate raw oxidation in athletes; RER in sedentary subjects; RER in athletes).

**Fig. 2:**
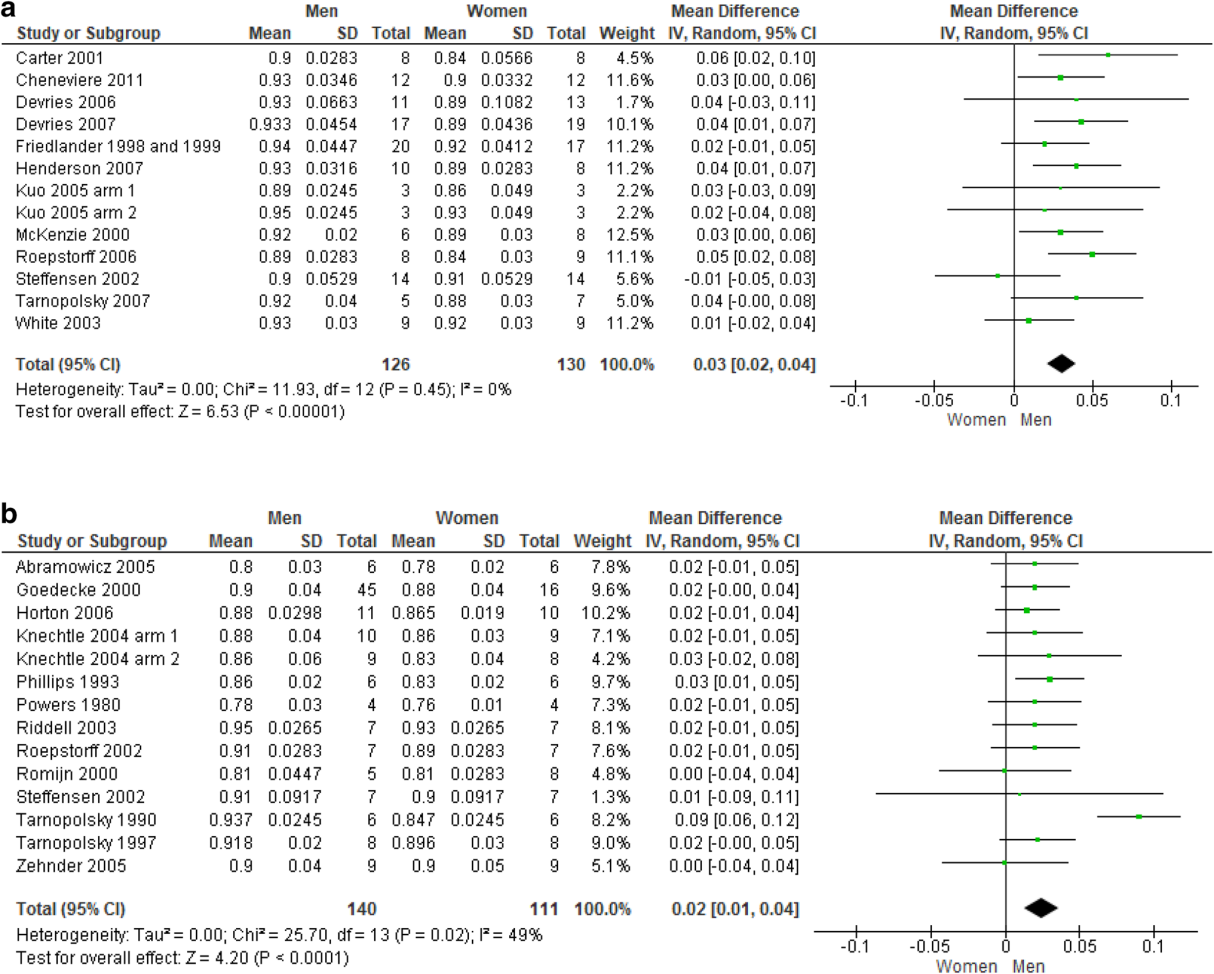
**a** Respiratory exchange ratio in sedentary subjects. **b** Respiratory exchange ratio in athletes

Meta-analytic aggregation for sex-based data in sedentary and athletic populations was completed for the following outcomes:

*RER* Figure 2 show RER results for the comparison between men and women during moderate aerobic exercise in sedentary (12 unique studies, 13 trials, 256 subjects) and athletic (13 unique studies, 14 trials, 251 subjects) populations, respectively. RER was found significantly higher in sedentary men than women (MD: + 0.03; 95% CI 0.02–0.04; *p* < 0.00001), at a moderate to large effect size (SMD: 0.69; 95% CI 0.42–0.97). Similarly, male athletes displayed a significantly higher RER than women (MD: + 0.02; 95% CI 0.01–0.04; *p* < 0.0001), at a moderate effect size (SMD: 0.57; 95% CI 0.30–0.83).

**Fig. 3:**
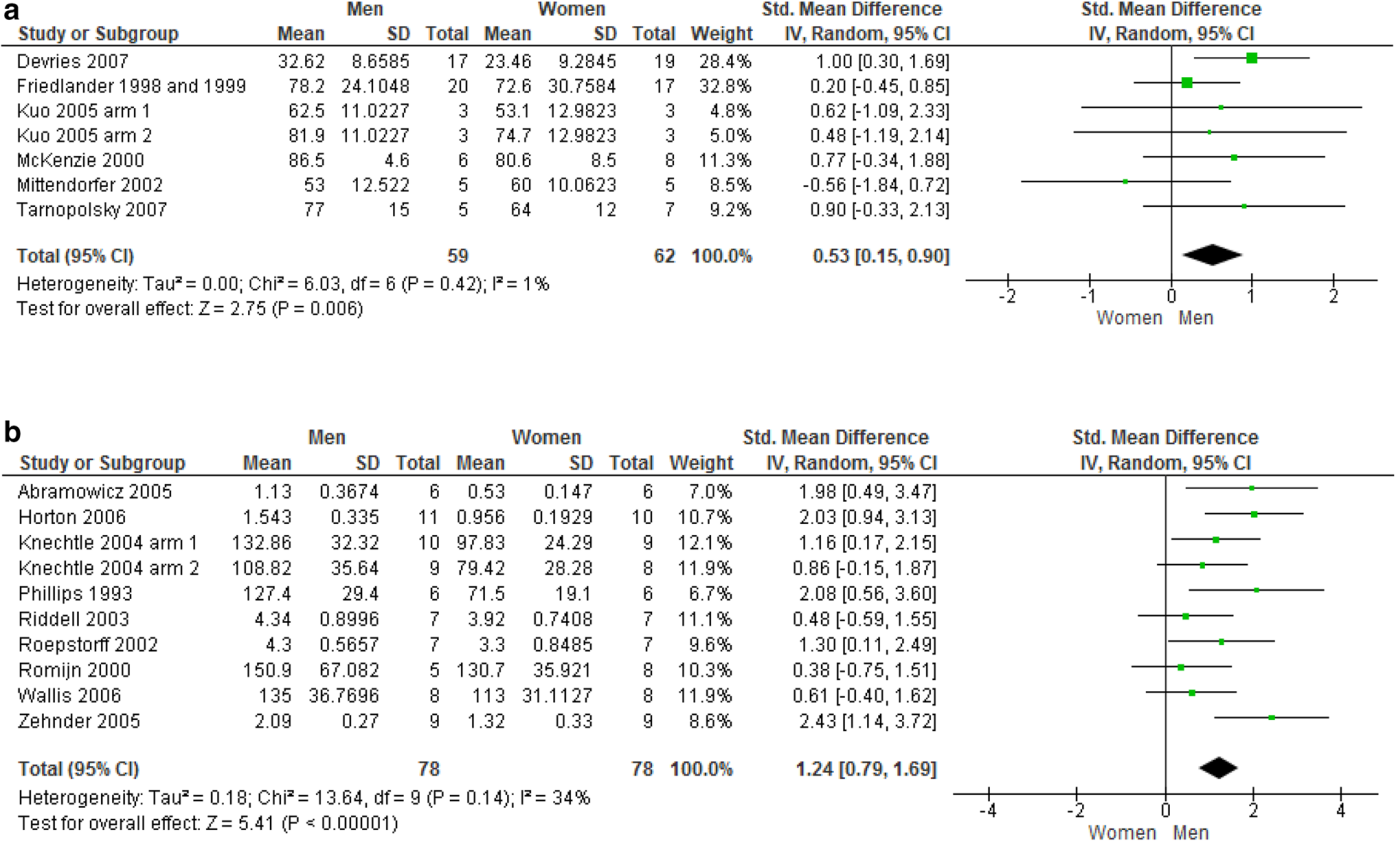
**a** Carbohydrate percent oxidation in sedentary subjects. **b** Carbohydrate raw oxidation in athletes

*Carbohydrate oxidation* Percent data pooling from six unique studies (7 trials, 121 subjects) revealed that sedentary men oxidize carbohydrates to a significantly greater extent than their female counterparts, at a moderate effect size (SMD: 0.53; 95% CI 0.15–0.90; *p* = 0.006; Fig. 3a). Similarly, the meta-analysis carried out by aggregating raw data from nine unique studies on athletes (10 trials, 156 subjects) showed that male athletes oxidize larger carbohydrates amount than female athletes, at a very large effect size (SMD: 1.24; 95% CI 0.79–1.69; *p* < 0.00001; Fig. 3b).

No meta-analyses could be performed for muscle glycogen utilization, as less than three studies shared the same outcome (percent contribution of muscle glycogen to total carbohydrate oxidation; muscle glycogen depletion following exercise; post-exercise muscle glycogen concentration).

**Fig. 4:**
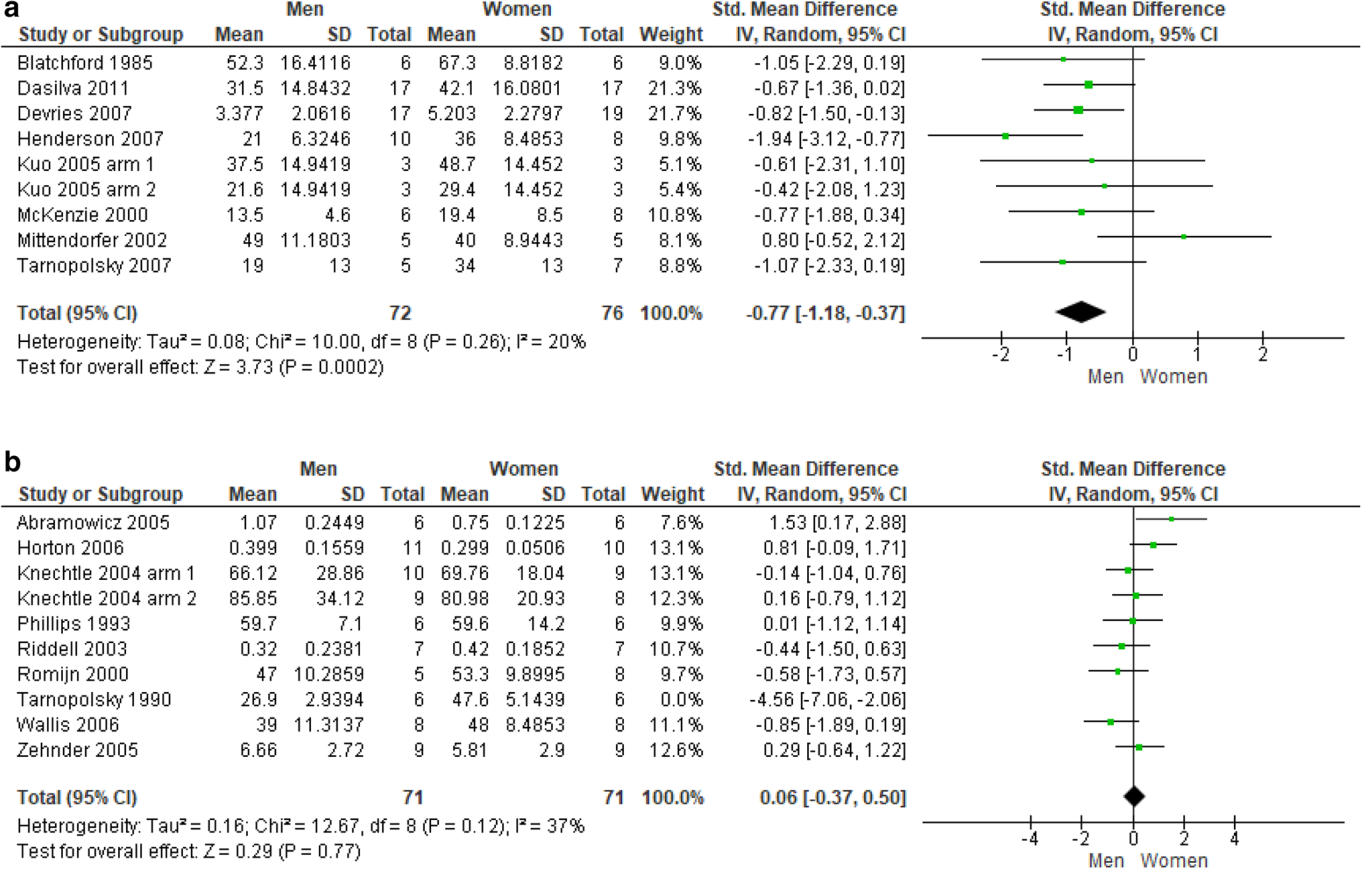
**a** Fat percent oxidation in sedentary subjects. **b** Fat raw oxidation in athletes

*Fat oxidation* Percent data pooling from eight unique studies (9 trials, 148 subjects) revealed that sedentary men oxidize fat sources to a significantly smaller extent than women, at a large effect size (SMD:  − 0.77; 95% CI  − 1.18  − 0.37; *p* = 0.0002; Fig. 4a). On the contrary, data pooling from nine unique studies conducted in athletic populations (10 trials, 154 subjects) showed no difference between male and female athletes in the pattern of fat oxidation. Due to excessive heterogeneity among the studies (I_2_ = 65%) brought by the study by Tarnopolsky et al. (1990), a *leave-one-out* approach was performed by deleting this study (SMD: 0.06; 95% CI  − 0.37, 0.50; *p* = 0.77; Fig. 4b).

*Protein oxidation* Data on protein oxidation could not be pooled as the two available studies (Horton et al. 1998; Lamont et al. 2001a) enrolled mixed samples including both sedentary and athletic subjects. With specific regard to athletic populations, aggregated data (percent oxidation) from two studies (Horton et al. 2006; Phillips et al. 1993, data not shown) showed a non-significant trend for larger protein oxidation in men than women (SMD: 0.65; 95% CI  − 0.06, 1.36; *p* = 0.07; 33 subjects).

**Fig. 5:**
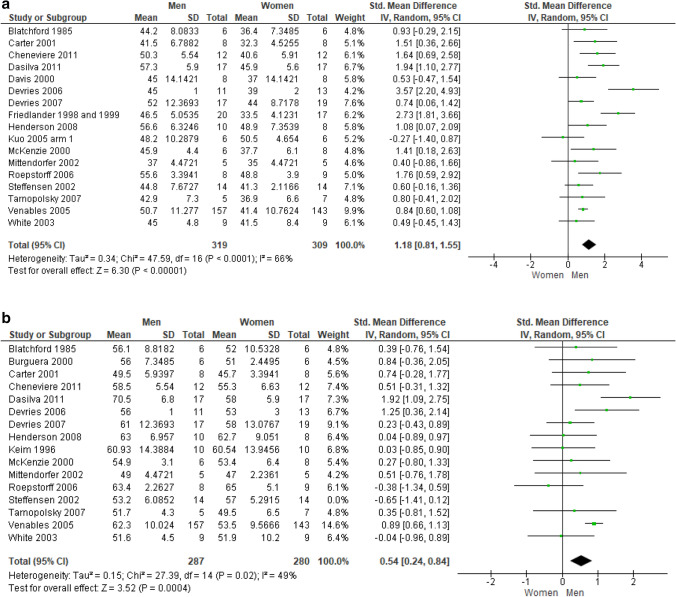
**a** Peak oxygen uptake in ml/min/kg in sedentary subjects. **b** Peak oxygen uptake in ml/min/kg normalized by lean body mass in sedentary subjects

*VO*_*2*_* peak* As expected, maximum oxygen consumption was found significantly higher in sedentary men than women, both when data were normalized to body weight (17 studies, 628 subjects; SMD: 1.18; 95% CI 0.81, 1.55; *p* < 0.00001; I^2^ = 66%, irreconcilable; Fig. 5a) or to lean body mass (16 studies, 595 subjects; SMD: 0.44; 95% CI 0.12, 0.77; *p* = 0.008). Due to excessive heterogeneity (I_2_ = 61%) among the studies where VO_2_ was normalized by lean body mass, a *leave-one-out* approach was performed by deleting the study by Steffensen et al. (2002) and correcting the pooled estimate (15 studies, 567 subjects; SMD: 0.54; 95% CI 0.24, 0.84; *p* = 0.0004; Fig. 5b).

**Fig. 6:**
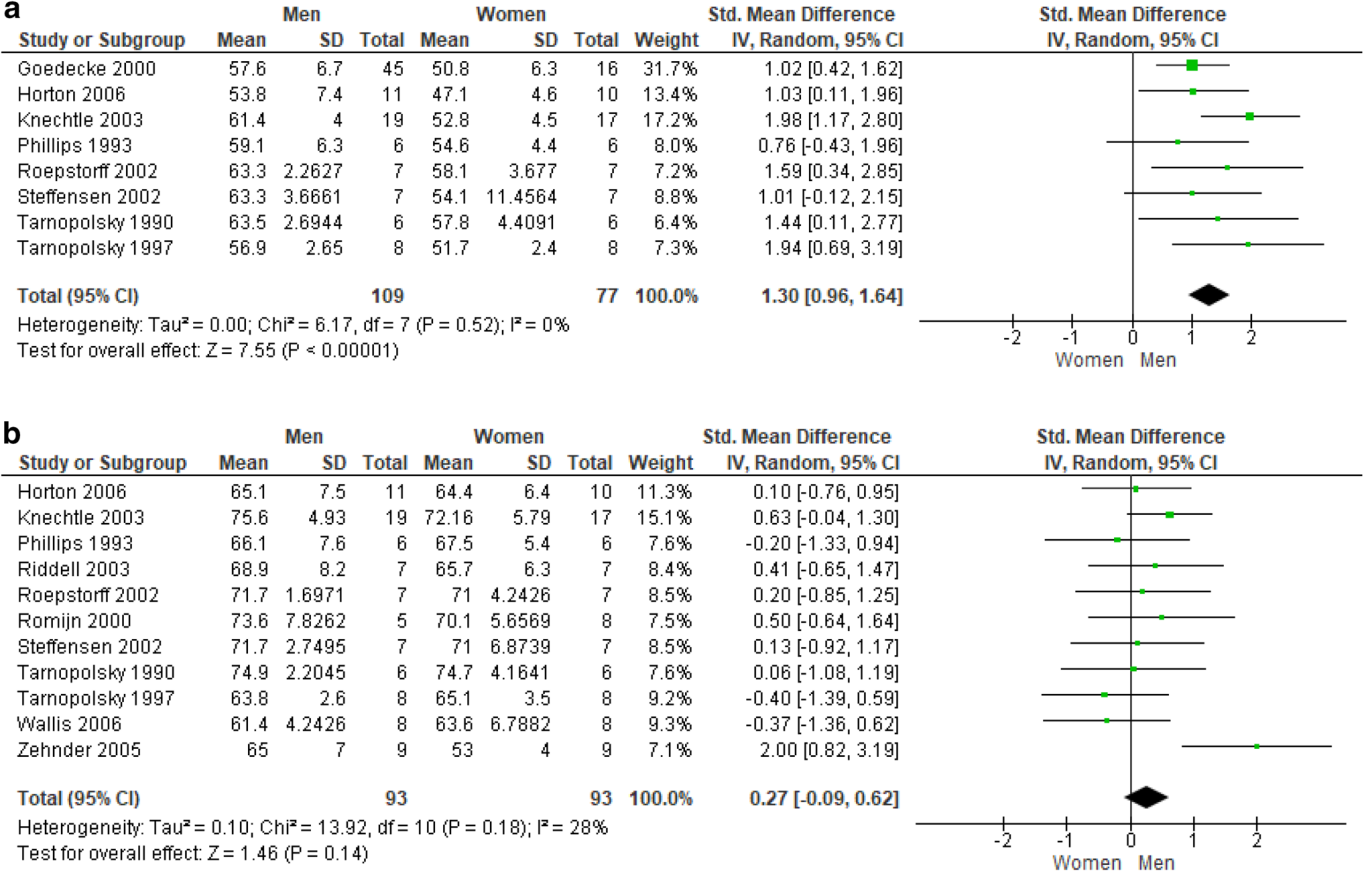
**a** Peak oxygen uptake in ml/min/kg in athletic subjects. **b** Peak oxygen uptake in ml/min/kg normalized by lean body mass in athletic subjects

While significantly higher VO_2_ peak in men was detected also in athletes with data normalized to body weight with a moderate quality of the evidence (8 studies, 186 subjects; SMD: 1.30; 95% CI 0.96, 1.64; *p* < 0.00001; Fig. 6a), no sex difference emerged after pooling data normalized to lean body mass (11 studies, 186 subjects; SMD: 0.27; 95% CI  − 0.09, 0.62; *p* = 0.14).

### Suggested mechanisms of sex-based differences in substrate utilization

**Fig. 7:**
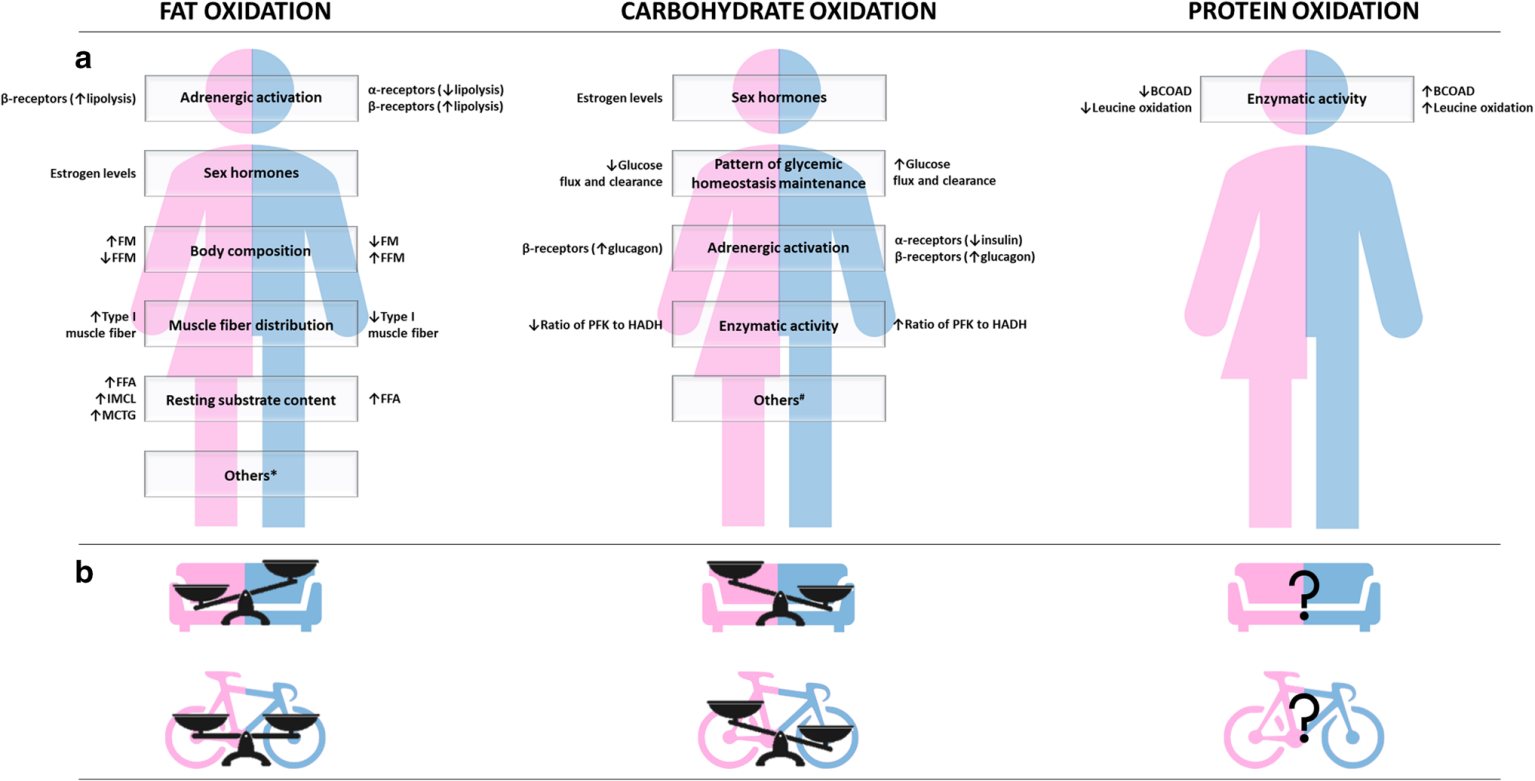
Graphical overview of the thematic analysis and graphical summary of the meta-analysis results. (A) The thematic analysis highlighted the most cited physiological contributors (boxes) to sex dimorphism in relation to fat, carbohydrate, and protein oxidation, during aerobic moderate-intensity exercise. Associated biological mechanisms that differ between women and men are specified on the left and on the right, respectively. (B) The meta-analysis confirmed sex-based differences in substrate utilization during aerobic moderate-intensity exercise. Sedentary women rely more on fat sources than sedentary men, although this was not confirmed in athletes. Men display greater reliance on carbohydrates than women, as observed both in sedentary (couch) and athletic (bike) populations. Paucity of studies on protein oxidation prevented meta-analytic aggregation, requiring further research. Others*: *enzymatic activity; gene expression; sex and adrenergic hormones’ interaction; cortisol; hormone-sensitive lipase; muscle capillarization; mRNA expression of genes; receptor availability/affinity.* others^#^: *resting substrate content; muscle fiber distribution: receptor availability/affinity; glucose recycling; hepatic glycogen sparing; muscle distribution*. Abbreviations: FM: fat mass; FFM: free-fat mass; FFA: free fatty acids; IMCL: intramyocellular lipid; MCTG: myocellular triacylglycerol; PFK: phosphofructokinase; HADH: 3-hydroxacyl-CoA dehydrogenase; BCOAD: branched-chain 2-oxoacid dehydrogenase

The main findings of the thematic analysis are graphically summarized in Fig. 7.

Among the 28 studies involving sedentary/recreationally active subjects, the main suggested mechanisms to explain sex dimorphism in fat utilization were differences in “adrenergic activation” (13 studies), “sex hormones” (10 studies), “body composition”, and “muscle fiber distribution” (5 studies). Less suggested mechanisms were: “resting substrate content” (i.e., baseline concentration; 2 studies), “different enzymatic activity” (1 study), “mRNA expression of genes associated with free fatty acid transport” (e.g., sarcolemmal free fatty acid transport protein and the membrane fatty acid binding protein; 1 study), “sex and adrenergic hormones’ interaction” (1 study), “cortisol concentration” (1 study), “higher content of and/or sensitivity to hormone-sensitive lipase (HSL) (1 study), muscle capillarization (1 study).

Regarding carbohydrate utilization, the main suggested mechanisms were differences in “sex hormones” (7 studies), “pattern of glycemic homeostasis maintenance” (i.e., the ability to regain/maintain glycemic homeostasis during exercise and post-exercise recovery; 3 studies), “adrenergic activation”, “enzymatic activity” (2 studies), “resting substrate content” (i.e., baseline concentration; 1 study), “muscle fiber distribution” (1 study), “receptor availability and affinity” (i.e., the ability of the sex hormonal milieu to modify the concentration of receptors and their ability to bind their specific ligands, modulating substrate utilization; e.g., insulin-binding receptors; 1 study), “mechanism of glucose recycling” (i.e., carbon recycling through gluconeogenesis from lactate, predominantly; 1 study), and “mechanism of hepatic glycogen sparing” (1 study).

Of the 17 studies regarding athletic populations, the most highlighted mechanisms regarding fat utilization in women and men were differences in “adrenergic activation” (4 studies), “muscle fiber distribution”, “resting substrate content” (3 studies), “sex hormones” (2 studies); “cortisol concentration” (1 study), and “higher content of and/or sensitivity to HSL (1 study). Sex differences regarding carbohydrate use during exercise were attributed to differences in “sex hormones” (4 studies), “adrenergic activation” (4 studies), “enzymatic activity” (3 studies), “pattern of glycemic homeostasis maintenance” (2 studies), and “muscle fiber distribution” (1 study).

Regarding protein metabolism, the thematic analysis was limited by the paucity of studies available on this topic. The three included studies (2 in sedentary subjects; 1 in athletes) converged on “different enzymatic activity” as a candidate mechanism for the observed sex differences in protein metabolism.

## Discussion

The present meta-analysis confirms that both sedentary and athletic males show preferential reliance on carbohydrates to sustain moderate aerobic exercise, while sedentary females rely more on lipids. By contrast, no difference in lipid oxidation rates was observed between male and female athletes, which is a novel finding of the present study.

Regarding the methodological quality of the studies reviewed, the risk for bias in the literature examined was rated as low to moderate. However, failure to clearly define inclusion and exclusion criteria for enrollment, limited statistical power, and absence of blinding procedures emerged as the main weaknesses in most of the included studies, thus introducing potential threats to the validity of the results reported by the individual studies.

### Sex-based differences in carbohydrate utilization

Overall, the pooled estimates confirmed the established knowledge that, compared with women, men rely significantly more on whole-body carbohydrate oxidation to sustain moderate-intensity aerobic exercise. This applied both to sedentary and athletic populations, as shown by the higher RER values and the higher percentage of carbohydrates oxidized to sustain the energetic demands. These results are in line with the literature on the topic outlining larger carbohydrate utilization in men by approximately 4–5% (Tarnopolsky 2000; Devries 2016).

Based on the magnitude of the effect size, reliance on carbohydrates appeared markedly larger among athletes than sedentary/recreationally active subjects. The findings on whole-body carbohydrate utilization are also in line with previous data regarding muscle substrate utilization, fiber types, and enzyme expression/activity. However, these data could not be pooled in our meta-analyses due to excessive methodological heterogeneity or paucity of studies sharing the same outcome measure. Friedlander and colleagues (1998) demonstrated reduced glucose flux and oxidation in women, as assessed by glucose rate of appearance, disappearance, and metabolic clearance. Based on this and other experimental evidence, women are generally reported to utilize 25–50% less muscle glycogen than matched men during moderate exercise (Tarnopolsky et al. 1990; Esbjörnsson-Liljedahl et al. 1999; Devries et al. 2006; Carter et al. 2001).

### Sex-based differences in lipid utilization

Interestingly, the common belief that women tend to rely on lipid sources during moderate aerobic exercise was confirmed in sedentary, but not in athletic populations. An athlete, by definition, is a person who has undertaken training or exercises to become proficient in physical activities such as competitive sports. Athletes are generally considered very fit compared with the general population of same sex and age group (Araújo and Scharhag 2016). The lack of difference between male and female athletes in lipid oxidation may be explained by the increased ability of male athletes to oxidize lipid sources per minute (maximal lipidic power) (Gonzalés-Haro et al. 2007). This adaptation might be due to their history of endurance training, compared with sedentary men, who preferentially oxidize carbohydrates.

While this finding has potential implications for training purposes, as male and female athletes exhibit similar fat oxidation rates, it is in discontinuity with a considerable body of literature that reported significantly larger reliance on lipid sources in women than men. Both experimental (Friedlander et al. 1998; Horton et al. 1998; Devries et al. 2007; Henderson et al. 2007; Tarnopolsky et al. 1990; 2007) and knowledge-synthesis works (Tarnopolsky 2000; Devries 2016) demonstrated a significantly lower RER in women, indicating higher whole-body fat oxidation. While the finding on RER was confirmed by our meta-analyses both in sedentary and athletic populations, it disagrees with previous studies that assessed regional substrate utilization, such as IMCL utilization and plasma FFA during endurance exercise. Indeed, both the systemic and leg FFA lipolytic response to aerobic exercise were not different between recreationally active men and women, as stated by Burguera and colleagues (2000). Likewise, FFA utilization was confirmed independent of sex also in athletes, after considering lean body mass differences (Romijn et al. 2000), in line with the findings of the present meta-analysis.

Data collected to examine the effect of sex on IMCL utilization patterns during moderate aerobic exercise are perhaps even more inconclusive. Some works failed to detect differences (White et al. 2003; Devries et al. 2007) or found larger (Roepstorff et al. 2002, 2006; Steffensen et al. 2002) or smaller (Zehnder et al. 2005) IMCL utilization in women than men. It has been suggested that methodological inconsistencies and training status differences, between participants within a trial, might contribute to these observed discrepancies (Devries 2016). Possibly for the same reasons, we could not complete a meta-analytical aggregation for FFA and IMCL data, thus preventing to quantify the magnitude of the differences reported in each individual study over a larger pooled sample.

Due to the paucity of sex-comparative studies on protein oxidation patterns during moderate aerobic exercise, no reliable and adequately powered meta-analyses could be performed. Therefore, previous findings from small-sized studies reporting lower oxidation of leucine (Phillips et al. 1993; McKenzie et al. 2000; Lamont et al. 2001a) and greater non-oxidative leucine disposal in women during endurance exercise (Lamont et al. 2001a) could not be confirmed.

### Main physiological mechanisms underpinning sex-based differences in substrate utilization

“Adrenergic activation” emerged as the most cited mechanism responsible for the larger reliance on lipid sources in both sedentary/recreationally active and athletic women. It was also frequently mentioned to partly explain the observed differences in carbohydrate utilization (ranked 3rd in sedentary/recreational, and 2nd in athletic populations). Tarnopolsky and colleagues (1990) suggested that, while exercise-induced changes in plasma growth hormone or glucagon concentrations could not explain the greater lipid utilization observed in women, the lower insulin and higher epinephrine concentrations seen in men could partially explain the greater glycogenolysis and glycogen utilization in this group.

Catecholamines are well known to stimulate hepatic glucose production through both increased glycogenolysis and gluconeogenesis. Activation of α-adrenoceptors by norepinephrine prompts an increase in blood glucose levels by reducing insulin secretion and glycogenolysis, whereas activation of β-adrenoceptors contributes to the rise of blood glucose levels by increasing glucagon and adrenocorticotropic hormone secretion (Chu et al. 1996; Horton et al. 2006).

Nevertheless, hormones’ biological activity depends not only on circulating concentrations, but also on receptor availability and sensitivity within the individuals. Women may be more sensitive to the lipolytic effects of catecholamines, whereas men may be more sensitive to the hormone’s glycolytic effects (Tarnopolsky et al. 1990). From a physiological standpoint, lipolysis in subcutaneous adipose tissue is mainly regulated by adrenergic mechanisms. As introduced earlier, in men, moderate exercise activates β1-(lipolysis stimulating) as well as α2-(lipolysis-inhibiting) adrenoceptors, whereas in women only β1-receptors are activated, thus supporting their favored kinetic profile of lipid mobilization (Boschmann et al. 2002).

Sex hormones, specifically ovarian hormones, were acknowledged as key contributors to the sex-based differences observed in substrate utilization (ranked 2nd for lipid utilization, in sedentary/recreational populations; 1st for carbohydrate, both in sedentary/recreational and athletic populations). In women, estrogen directly reduces carbohydrate utilization due to a marked hepatic glycogen sparing effect and insulin-mediated storage, thus indirectly shifting metabolism toward lipids, mainly via FFA mobilization and oxidation (Friedlander 1998; Horton et al. 1998; Carter et al. 2001). Additionally, evidence indicates that women, in comparison to men, have more and larger adipocytes in the gluteal region, which display greater sensitivity to lipolytic agents, such as sex hormones and catecholamines, compared to adipose cells in other sites. Consequently, women display more pronounced regional differences in the hormonal regulation of lipolysis than men during exercise (Blatchford et al. 1985; Arner et al. 1990).

Although relatively minor, compared to sex hormones and adrenergic mechanisms, “muscle fiber distribution” was another factor that emerged from our thematic analysis. Several included studies partly explained sex dimorphism in lipid oxidation based on the established evidence that women have a higher percentage of type I highly oxidative low glycolytic fibers, whereas men display a significantly higher proportion of type II highly glycolytic low oxidative fibers (Steffensen et al. 2002). The typical fiber distribution in women is type I > type IIA > type IIX compared to men with type IIA > type I > type IIX (Staron et al. 2000). This evidence would explain why women can oxidize more fat in their muscles, exhibiting reduced muscle fatigability during moderate exercise, while men’s metabolism is shifted toward glycolysis to obtain energy (Tarnopolsky et al. 1990; Zierath and Hawley 2004).

Finally, resting substrate content emerged as another mechanism mediating the sex-based differences in substrate utilization. It has been claimed that the higher lipolysis rates in women may partly relate to the larger availability of lipid substrates to support endurance exercise. While women have greater storages of IMCL (Roepstorff et al. 2002; Devries et al. 2007), their greater capacity to use this substrate is still debated, as some studies failed to detect sex differences (White et al. 2003; Devries et al. 2007). However, women have a greater percentage of IMCL in direct contact with mitochondria after a bout of endurance exercise compared with men, which suggests that they may have a greater capacity to use IMCL (Devries 2016) and, thus, a metabolic advantage for endurance when exercising at matched relative intensities (Boschmann et al. 2002; Tarnopolsky et al. 2007).

Women were found to rely more on fat as energy source, thereby using less carbohydrate, amino acid, and protein compared with male exercisers (Phillips et al. 1993; Lamont et al. 2001a). The precise mechanism for the sex difference in protein utilization is still debated. However, the percent activation of hepatic branched-chain 2-oxoacid dehydrogenase appears higher in men, in line with the findings by McKenzie and colleagues (2000). Given the paucity of data on the protein kinetics of men and women during moderate endurance exercise, further sex-comparative studies on protein metabolism are needed.

### Study limitations

A number of potential limitations to the validity of the pooled estimates, outlined in the present review, should be acknowledged. First, the frequent report of mixed samples (sedentary and recreationally active individuals) in most of the studies that did not focus on athletes. Relatedly, all the studies included in this meta-analysis enrolled young adults (aged 18–35 years**)**, thus making our results not generalizable to all age groups. Second, 10% of the pertinent studies had to be excluded from the analysis, as they enrolled mainly men as participants. This confirms the marked sex bias affecting the research on strategies intended to improve exercise performance and/or health (Devries 2016; Cugusi et al. 2019). Investigators tend to exclude female participants due to the potential influence of fluctuating ovarian hormones throughout the menstrual cycle and its impact on the outcomes of interest. Indeed, when female participants are included in the studies, a poor consideration and characterization of the ovarian hormonal status, menstrual cycle phases, and use of oral contraceptives can be observed, leading to lack of information and inherent mixed female population (Elliott-Sale et al. 2021). Such heterogeneity and lack of reporting may be a potentially limiting factor for the validity of the pooled estimates here obtained. Third, neither diet assessment nor control (prior to exercise testing) were consistently reported by the studies, introducing a certain degree of methodological heterogeneity that may have limited the accuracy of some of the estimates here outlined. Fourth, another element that potentially limits the strength of the findings in athletic populations relates to the exclusion of studies that involved nutritional interventions or supplementation. For those works that planned such interventions, we only considered data from the study arm (if any) where participants were given plain water. Finally, while the range between 45 and 65% of peak aerobic capacity is well accepted to resemble moderate-intensity aerobic exercise in untrained individuals, this may not apply to endurance-trained subjects who may display high anaerobic threshold, requiring a higher intensity (i.e., 70–75% of VO_2_ peak) to match "moderate” aerobic exercise.

### Conclusions and future directions

Meta-analytical aggregations confirmed the occurrence of sex-based differences in fuel utilization during moderate aerobic exercise. Men display higher RER and, accordingly, greater reliance on carbohydrates, whereas sedentary women rely more on fat sources. However, the latter finding was not confirmed in athletes, which is a novel aspect of the present study that requires future tailored investigations. Overall, carbohydrate and lipid kinetics of utilization, during endurance exercise, have been extensively investigated. As emerged, this does not apply to protein metabolism, for evident paucity of data, requiring further research.

The analysis of the main suggested physiological mechanisms related to sex-based difference in substrate utilization during exercise has highlighted the need for mechanistically driven research. Future investigation should not only focus on whole-body substrate utilization patterns, but also include organ-, histological- and cellular-level outcomes, the latter being frequently neglected for lipid and protein metabolism both in sedentary and athletic populations. Moreover, the nutritional status (e.g., body composition, food intake, energy expenditure, pre-testing diet) should be taken into proper consideration since the planning stage of the study, as it can affect substrate metabolism and resting substrate storage.

To reduce the overall heterogeneity of the existing body of literature on the topic and to improve our understanding of the sex-based differences in substrate utilization, future studies should: (a) consider the diversity and complexities associated with female endocrinology across the lifespan (e.g., menstrual cycle, hormonal contraceptive use, pregnancy, menopause), (b) effectively adapt experimental designs to incorporate female-specific considerations, and (c) clearly characterize female populations included in the study, using the appropriate nomenclature.

Therefore, we recommend that upcoming studies involving women in sport and exercise science adhere to the most recent working guide for standards of practice on the topic (Elliott-Sale et al. 2021). Moreover, to assess the menstrual cycle status and phases, we recommend following the methodological guidance by Janse de Jonge et al. (2019).

Overall, these implementations will likely provide useful information for tailored nutritional and exercise interventions for men and women, addressed toward both the maintenance of good health status and performance improvement.

## Abbreviations

CI: Confidence interval
FFA: Free fatty acids
HSL: Hormone-sensitive lipase
I^2^: Inconsistency test
IMCL: Intramyocellular lipid
MD: Mean difference
MeSH: Medical Subject Heading
NIH: National Institutes of Health
O_2_: Oxygen
RER: Respiratory exchange ratio
SD: Standard deviation
SMD: Standardized mean difference
VO_2_: Volume of oxygen
