# Correction to: Analysis of sex-based differences in energy substrate utilization during moderate-intensity aerobic exercise

**DOI:** 10.1007/s00421-022-04961-z

**Published:** 2022-05-05

**Authors:** Antonella Cano, Lucia Ventura, Gianluca Martinez, Lucia Cugusi, Marcello Caria, Franca Deriu, Andrea Manca

**Affiliations:** 1 Department of Biomedical Sciences, University of Sassari, Viale S. Pietro 43/b, 07100 Sassari, Italy; 2 Unit of Endocrinology, Nutritional and Metabolic Disorders, AOU Sassari, Sassari, Italy

## Correction to: European Journal of Applied Physiology (2022) 122:29–70 10.1007/s00421-021-04802-5

The original version of this article unfortunately contained a mistake. Acknowledgements section should read as

This work was supported by the Fondo di Ateneo per la Ricerca 2019 (FAR 2019) dell’Università degli Studi di Sassari.

